# Mucosal and Systemic Responses to Severe Acute Respiratory Syndrome Coronavirus 2 Vaccination Determined by Severity of Primary Infection

**DOI:** 10.1128/msphere.00279-22

**Published:** 2022-11-02

**Authors:** Mohammad M. Sajadi, Amber Myers, James Logue, Saman Saadat, Narjes Shokatpour, James Quinn, Michelle Newman, Meagan Deming, Zahra Rikhtegaran Tehrani, Laurence S. Magder, Maryam Karimi, Abdolrahim Abbasi, Mike Shlyak, Lauren Baracco, Matthew B. Frieman, Shane Crotty, Anthony D. Harris

**Affiliations:** a Baltimore VA Medical Center, VA Maryland Health Care System, Baltimore, Maryland, USA; b Institute of Human Virology, University of Maryland School of Medicine, Baltimore, Maryland, USA; c Department of Microbiology and Immunology, University of Maryland School of Medicine, Baltimore, Maryland, USA; d Center for Infectious Disease and Vaccine Research, La Jolla Institute for Immunology, La Jolla, California, USA; e Department of Epidemiology and Public Health, University of Maryland School of Medicine, Baltimore, Maryland, USA; f Department of Medicine, Division of Infectious Diseases and Global Public Health, University of California, San Diego (UCSD), La Jolla, California, USA; University of Arizona

**Keywords:** SARS-CoV-2, vaccination, systemic response, mucosal immunity, IgA, IgG

## Abstract

With much of the world infected with or vaccinated against severe acute respiratory syndrome coronavirus 2 (commonly abbreviated SARS-CoV-2; abbreviated here SARS2), understanding the immune responses to the SARS2 spike (S) protein in different situations is crucial to controlling the pandemic. We studied the clinical, systemic, mucosal, and cellular responses to two doses of SARS2 mRNA vaccines in 62 individuals with and without prior SARS2 infection that were divided into three groups based on antibody serostatus prior to vaccination and/or degree of disease symptoms among those with prior SARS2 infection: antibody negative (naive), low symptomatic, and symptomatic. Antibody negative were subjects who were antibody negative (i.e., those with no prior infection). Low symptomatic subjects were those who were antibody negative and had minimal or no symptoms at time of SARS2 infection. Symptomatic subjects were those who were antibody positive and symptomatic at time of SARS2 infection. All three groups were then studied when they received their SARS2 mRNA vaccines. In the previously SARS2-infected (based on antibody test) low symptomatic and symptomatic groups, reactogenic symptoms related to a recall response were elicited after the first vaccination. Anti-S trimer IgA and IgG titers, and neutralizing antibody titers, peaked after the 1st vaccination in the previously SARS2-infected groups and were significantly higher than for the SARS2 antibody-negative group in the plasma and nasal samples at most time points. Nasal and plasma IgA antibody responses were significantly higher in the low symptomatic group than in the symptomatic group at most time points. After the first vaccination, differences in cellular immunity were not evident between groups, but the activation-induced cell marker (AIM^+^) CD4^+^ cell response correlated with durability of IgG humoral immunity against the SARS2 S protein. In those SARS2-infected subjects, severity of infection dictated plasma and nasal IgA responses in primary infection as well as response to vaccination (peak responses and durability), which could have implications for continued protection against reinfection. Lingering differences between the SARS2-infected and SARS2-naive up to 10 months postvaccination could explain the decreased reinfection rates in the SARS2-infected vaccinees recently reported and suggests that additional strategies (such as boosting of the SARS2-naive vaccinees) are needed to narrow the differences observed between these groups.

**IMPORTANCE** This study on SARS2 vaccination in those with and without previous exposure to the virus demonstrates that severity of infection dictates IgA responses in primary infection as well as response to vaccination (peak responses and durability), which could have implications for continued protection against reinfection.

## INTRODUCTION

Although several recent studies have shown protection from reinfection among those with severe acute respiratory syndrome coronavirus 2 (commonly abbreviated SARS-CoV-2; abbreviated here SARS2) infection who are subsequently vaccinated (1, 2), controversy exists as to the degree of protection from reinfection in those with prior SARS2 infection, as well as the role for vaccination and boosters in this population. The heterogeneous course of primary infection, which can span from asymptomatic infection to intensive care unit (ICU) admission and death, is linked to differing immune responses. Understanding the primary immune responses to natural infection and vaccination, as well as the secondary responses in those previously infected or vaccinated, is key to understanding the protection. Given the recent findings of a decrease in durability of protection after vaccination (3), this has become even more urgent.

Early in the pandemic, it was noted that severity of infection was linked to higher IgG titers. For example, it was shown that hospitalized patients with COVID-19 with ICU admission have higher IgG responses to spike (S) protein (4) than non-ICU hospitalized patients and that hospitalized patients have higher IgG titers to spike than nonhospitalized patients (5, 6). Vaccination after primary infection induces hybrid immunity, but it is not known if differences in course of primary infection affect the secondary responses or have any implications for protection.

The current study was undertaken to investigate the primary responses in COVID-19-naive vaccinees defined as antibody (Ab) negative prior to vaccination, as well as secondary responses in those with previous COVID-19, defined as antibody positive prior to vaccination with various severities of initial infection.

## RESULTS

### The first vaccination elicited elevated symptoms (reactogenicity) in SARS2-infected individuals.

A total of 3,816 health care workers (HCW) were enrolled in a SARS2 serosurvey study (7), of whom 151 were randomly contacted as part of this study, and 67 volunteers with known SARS2 serostatus enrolled. Of the 67, 62 received both doses of Comirnaty (Pfizer) or Spikevax (Moderna) COVID-19 vaccine (4 had dropped out prior to the second vaccination, and 1 was diagnosed with COVID-19 before the second vaccination). The 62 who received both vaccines were divided into the following groups: 19 antibody negative, 17 antibody positive and low symptomatic (history of being asymptomatic or minimally symptomatic with a total of 2 or fewer days of work missed due to initial SARS2 infection), and 26 symptomatic antibody positive (history of 3 or more days of work missed due to infection, with or without hospitalization after initial infection) (Table 1).

**TABLE 1:** Study population baseline characteristics

Parameter	Ab negative (*n* = 19)	Ab positive (*n* = 43)		*P* value^*a*^
		Low symptomatic (*n* = 17)	Symptomatic (*n* = 26)	
Age (yrs)				
Median	42	39	38	0.39
Range	29–62	25–72	23–59	
Sex (no. [%])				
Male	5 (26)	4 (24)	3 (12)	0.49
Female	14 (74)	13 (76)	23 (88)	
Race/ethnicity (no. [%])^*b*^				
Black or African American	2 (11)	5 (29)	5 (19)	0.31
White or Caucasian	14 (74)	9 (53)	18 (69)	0.56
Asian	3 (16)	3 (18)	3 (14)	1.0
Hispanic or Latino	0 (0)	0 (0)	0 (0)	1.0
Vaccine (no. [%])				
Pfizer	11 (58)	7 (41)	13 (50)	0.58
Moderna	8 (42)	10 (59)	13 (50)	
Days of missed work				
Median	NA^*c*^	0	10	NA
Range	NA	0–2	3–34	NA
Hospitalization (no. [%])	NA	0 (0)	4 (15)	NA
Mo from SARS2 PCR^+^ to vaccination				
Median (percent PCR tested)	NA	9.0 (41)	8.0 (73)	NA
Range	NA	6.5–9.7	6.0–10.3	NA
Mo from SARS2 IgG+ to vaccination				
Median	NA	6.1	6.1	NA
Range	NA	4.8–7.1	3.8–7.2	NA
Time from baseline sample to vaccination				
Median	0	2	5	0.03
Range	0–22	0–27	0–30	NA

a *P* value reports analysis between antibody-negative and antibody-positive groups.

b Race/ethnicity data derived from self-report. This is of research interest because people of different races/ethnicities may react differently to vaccine.

c NA, not applicable.

**FIG 1:**
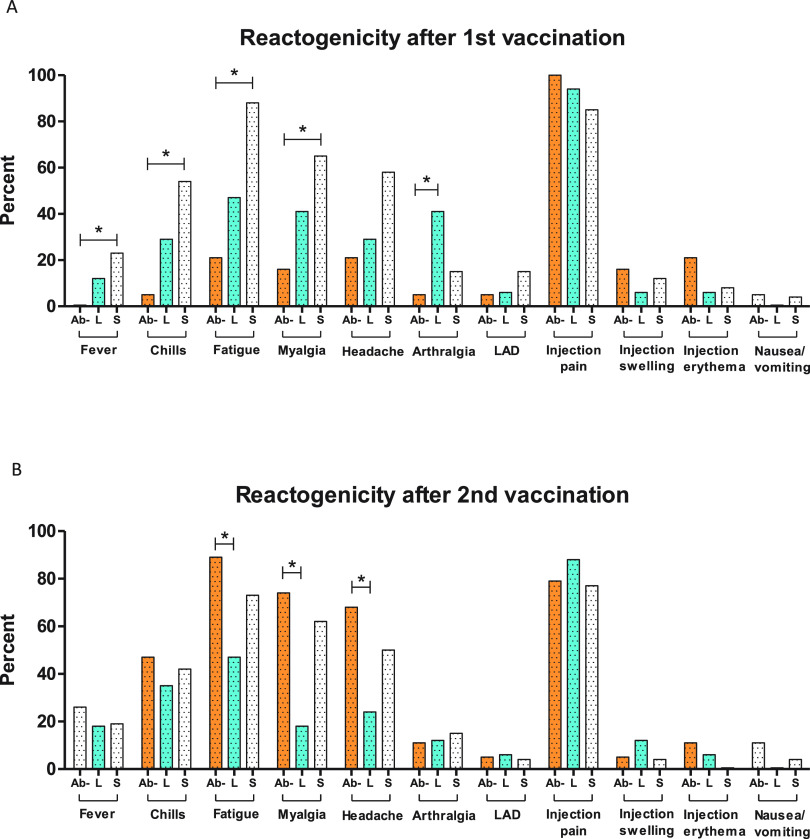
Reactogenicity after first and second vaccination. Percent reported symptoms after the first (A) and second (B) vaccinations is shown on the *y* axis. Ab-negative (Ab-), low symptomatic (L), and symptomatic (S) groups are shown in orange, blue, and white, respectively. LAD, lymphadenopathy. Each symptom was assessed by 2-tailed Fisher’s exact test between the Ab-negative and the low symptomatic or symptomatic group, with *P* values of <0.05 being considered significant and shown with asterisks.

After the first vaccination, individuals in the low symptomatic and symptomatic antibody-positive groups had higher rates of vaccine-related systemic symptoms than those in the antibody-negative group, some of which were statistically significant between the symptomatic and Ab-negative groups (Fig. 1). After the second vaccination, the Ab-negative group had an increase in reactogenicity compared to the first vaccination, and any individual differences from the symptomatic group were no longer statistically significant. However, after the second vaccination, the low symptomatic group had less fatigue and fewer reports of myalgias and headaches than the Ab-negative group (Fig. 1). After the first and second vaccinations, the low symptomatic group had less reactogenicity than the symptomatic group in nearly every category, and although the individual categories did not reach statistical significance, the low symptomatic group had fewer systemic symptoms than the symptomatic group when considering the number of systemic symptoms reported in each category of 1 more (71% versus 96%) or 2 or more (41% versus 77%) systemic symptoms (*P* = 0.03 and *P* = 0.03, respectively).

### SARS2-infected individuals had higher titers of IgG nasal and plasma antibodies than SARS2-naive individuals.

**FIG 2:**
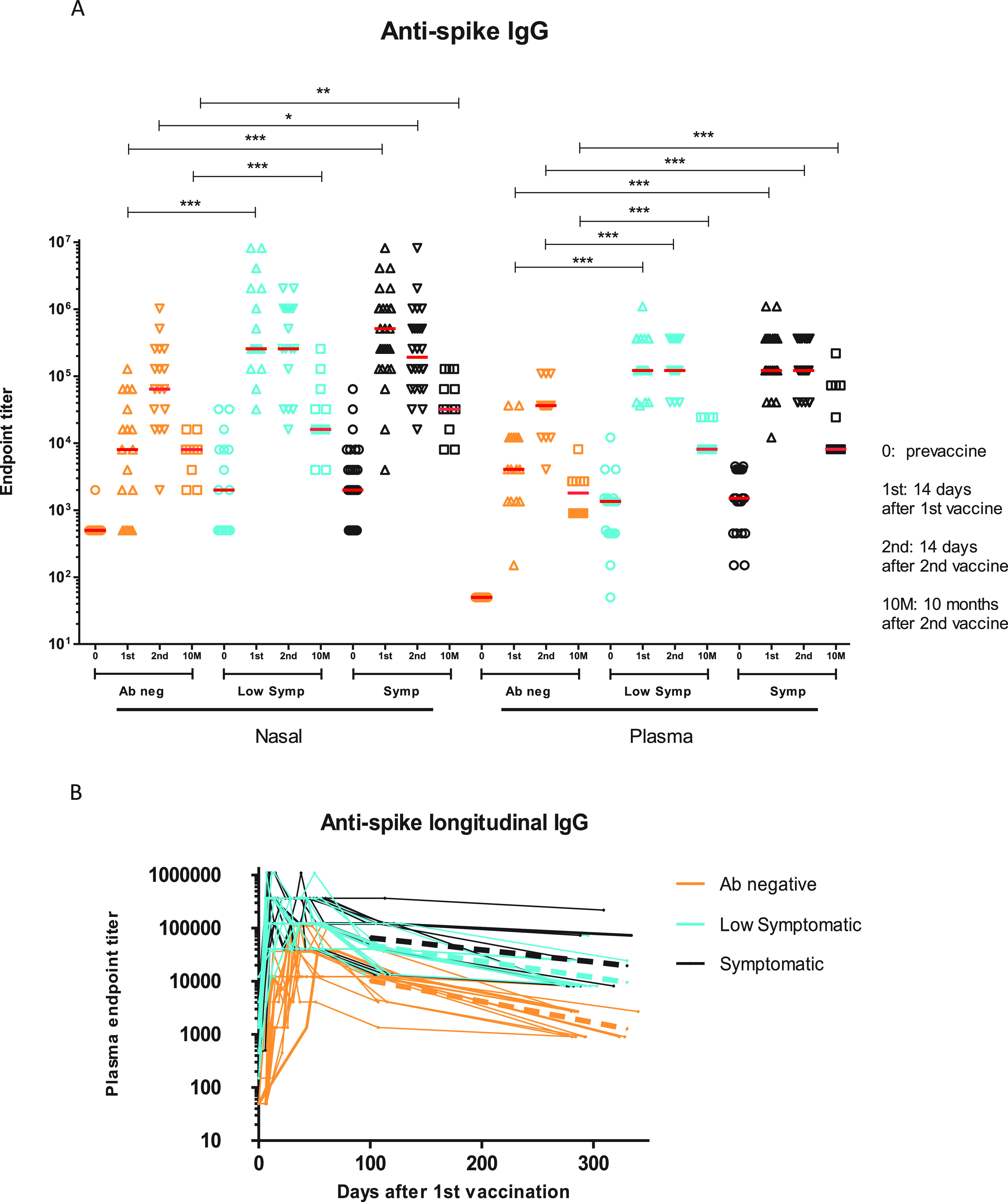
Nasal and systemic IgG ELISA responses to SARS2 S trimer. (A) IgG endpoint titers were measured at baseline, 14 days after the 1st vaccination, 14 days after the 2nd vaccination, and 10 months after the 2nd vaccination. Horizontal red lines represent median values. (B) IgG endpoint titers measured at all time points until 10 months after the second vaccination. Dotted lines represent decay slopes for each cohort between months 3 and 10. The limit of detection >(LOD) for the nasal IgG ELISA is 1:500, and that for the plasma IgG ELISA is 1:50 (dotted black lines). Ab-negative, low symptomatic, and symptomatic groups are shown in orange, blue, and black, respectively. The *y* axis represents reciprocal endpoint titer to SARS2 S trimer in a logarithmic scale. *, *P* ≤ 0.05; **, *P* ≤ 0.01; ***, *P* ≤ 0.001; ****, *P* ≤ 0.0001.

At baseline, the antibody-negative groups had undetectable nasal and plasma endpoint titers of IgG antibodies to the SARS2 spike trimer, whereas these could be detected to various degrees for the low symptomatic and symptomatic groups (Fig. 2A). Specifically, prior to vaccination, nasal IgG against S was detected in 53% of the low symptomatic group and 65% of the symptomatic group. Nasal and plasma IgG S trimer endpoint titers continued to increase after the 2nd vaccination in the Ab-negative group, while they plateaued after the first vaccination in the low symptomatic and symptomatic groups (Fig. 2A). IgG S trimer endpoint titers after each vaccination were higher in the low symptomatic and symptomatic groups than in the Ab-negative group (Fig. 2A), and this difference continued in the plasma and nose until the study completion, up to 10 months after the second vaccination (Fig. 2B). At the last time point the median plasma endpoint titer for the antibody-negative group was 2,700, statistically lower than for the low symptomatic (8,100) and symptomatic (8,100) groups (*P* ≤ 0.001 and *P* ≤ 0.001, respectively). There was no significant difference in the slope of the decline of plasma IgG binding titers between 3 months after the second dose and up to 10 months after the second dose in the Ab-negative (−0.118 log titer/month) group compared to the low symptomatic (−0.091 log titer/month) and symptomatic (−0.069 log titer/month) groups (*P* = 0.43 and *P* = 0.32, respectively) (Fig. 2B). Comparing Moderna versus Pfizer recipients within each group, we noted larger peak plasma IgG anti-S binding responses in SARS2-infected volunteers at 14 days after the first Moderna vaccine (*P* = 0.0056) but not at any other time points (see Fig. S1A in the supplemental material; Table S1).

10.1128/msphere.00279-22.1 FIG S1 Comparison of plasma binding and neutralization titers by vaccination group. Antibody negative, low symptomatic, symptomatic, and SARS2-infected (combined low symptomatic/symptomatic) groups are shown in orange, blue, and black, respectively. The *y* axis represents reciprocal endpoint IgG titer (or ID_99_ neutralization titer) to SARS2 spike trimer in a logarithmic scale. Intragroup binding and neutralization titers at the different time points were analyzed by two-tailed Mann-Whitney test. M, Moderna (triangles); P, Pfizer (circles). 1^st^, 14 days after first vaccination; 2^nd^, 14 days after second vaccination. *, *P* ≤ 0.05; **, *P* ≤ 0.01; ***, *P* ≤ 0.001; ****, *P* ≤ 0.0001. Download FIG S1, TIF file, 0.1 MB. Copyright © 2022 Sajadi et al. 2022 Sajadi et al. https://creativecommons.org/licenses/by/4.0/ This content is distributed under the terms of the Creative Commons Attribution 4.0 International license.

### SARS2-infected individuals in the low symptomatic group had higher IgA nasal and plasma antibodies than SARS2-infected individuals in the symptomatic group.

**FIG 3:**
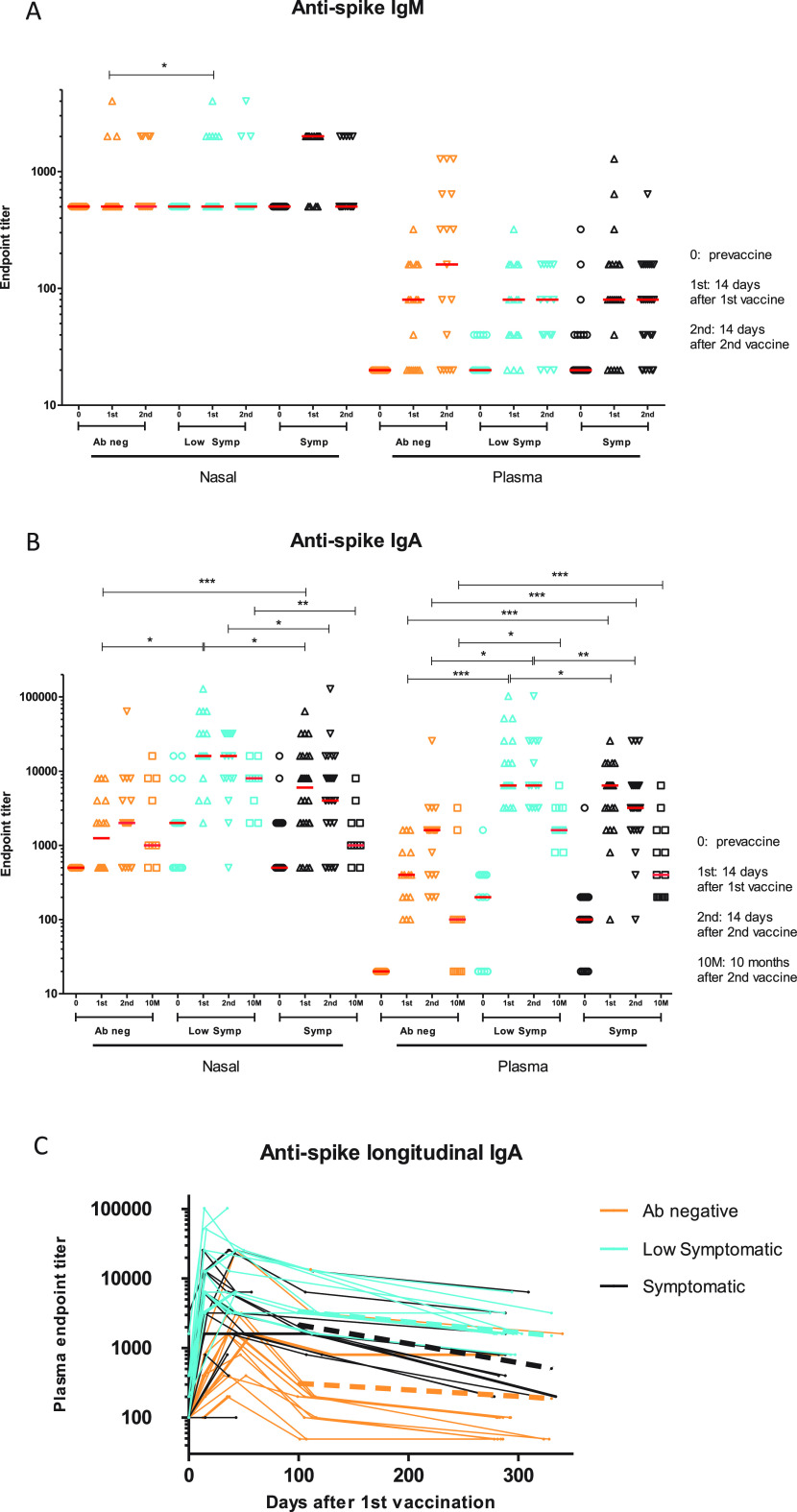
Nasal and systemic IgM and IgA ELISA responses to SARS2 S trimer. (A) IgM endpoint titers were measured at baseline, 14 days after the 1st vaccination, and 14 days after the 2nd vaccination. Horizontal red lines represent median values. (B) IgA endpoint titers were measured at baseline, 14 days after the 1st vaccination, 14 days after the 2nd vaccination, and 10 months after the 2nd vaccination. Horizontal red lines represent median values. (C) IgA endpoint titers measured at all time points until 10 months after the second vaccination. The low symptomatic group had a smaller slope of decline for IgA binding titers (−0.045 log titer/month) than the symptomatic group (−0.082 log titer/month) but not the antibody-negative group (−0.028 log titer/month) (*P* = 0.04 and *P* = 0.59, respectively). Dotted lines represent decay slopes for each cohort between months 3 and 10. The LOD for the nasal IgM ELISA is 1:500, that for nasal IgA is 1:500, that for plasma IgM is 1:20, and that for plasma IgA is 1:100 (dotted black lines). Ab-negative, low symptomatic, and symptomatic groups are shown in orange, blue, and black, respectively. The *y* axis represents reciprocal endpoint titer to SARS2 S trimer in a logarithmic scale. *, *P* ≤ 0.05; **, *P* ≤ 0.01; ***, *P* ≤ 0.001; ****, *P* ≤ 0.0001.

At baseline, the antibody-negative groups had undetectable nasal and plasma endpoint titers of IgM and IgA antibodies to the SARS2 spike trimer, whereas these could be detected to various degrees in the low symptomatic and symptomatic groups (Fig. 3A and B). Specifically, prior to vaccination, nasal IgA against S was detected in 47% of the low symptomatic group and 42% of the symptomatic group. Plasma IgA S trimer endpoint titers continued to increase after 2nd vaccination in the Ab-negative group, while they plateaued after the first vaccination in the low symptomatic and symptomatic groups (Fig. 3B). Nasal and plasma IgA S trimer endpoint titers at each time point were significantly higher in the low symptomatic group than in the symptomatic group at most time points, and this difference continued in the plasma and nose until the study completion, up to 10 months after the second vaccination (Fig. 3B and C, Table S1A and Table S1B). Between 3 months after the second dose and up to 10 months after the second dose, the low symptomatic group had a smaller slope of decline for IgA binding titers (−0.045 log titer/month) than the symptomatic group (−0.082 log titer/month) but not the antibody-negative group (−0.028 log titer/month) (*P* = 0.04 and *P* = 0.59, respectively) (Fig. 3B and Table S1A). Peak nasal IgA titers were associated with lower age and male sex (Table S1A and S1B). Days of reported symptoms during SARS2 infection correlated with peak serum IgA titers (*P* = 0.04) but not peak nasal IgA titers (*P* = 0.07).

10.1128/msphere.00279-22.10 TABLE S1 (A) Relationship between categorical predictors and select immunologic measures (Wilcoxon Test); (B) Spearman correlations between quantitative predictors and antibody durability/peak among those with SARS2 infection prior to vaccination. Download Table S1, TIF file, 0.1 MB. Copyright © 2022 Sajadi et al. 2022 Sajadi et al. https://creativecommons.org/licenses/by/4.0/ This content is distributed under the terms of the Creative Commons Attribution 4.0 International license.

In the nasal samples, the median IgG/IgA ratio for total nonvaccine specific antibody was 0.76, compared to 7.97 in the plasma (*P* < 0.0001), and at baseline in the low symptomatic and symptomatic groups, the median SARS2-specific IgG/IgA ratio was 2.0, compared to 7.5 in the plasma (*P* < 0.0001) (Fig. S2B). After the first vaccination, the median SARS2 specific IgG/IgA increased to 64 in the nasal samples, compared to 23.73 in plasma (*P* = 0.04) (Fig. S2B). Nasal spike-specific antibody carrying the secretory component (potentially representing either IgA or IgM, as IgG does not carry a secretory component) was not seen at baseline but was detected in a majority of the low symptomatic and symptomatic groups after the first vaccination (Fig. S2C).

10.1128/msphere.00279-22.2 FIG S2 Nasal IgA binding and secretory antibody in vaccinees with history of SARS2 infection. (A) Log-transformed peak nasal IgA (14 days after first vaccination) plotted against days of work missed during primary infection and analyzed by linear regression. (B) IgG/IgA ratios measured in nasal samples and plasma samples in combined low symptomatic and symptomatic groups. Total IgG/IgA measured in nasal and plasma samples from various time points and total IgG/IgA and anti-spike trimer IgG/IgA measured at baseline and day 14 after the first vaccination are shown. (C) Anti-spike antibody carrying the secretory protein was measured in samples at baseline and 14 days after the first vaccination. For calculations of IgG/IgA, sample pairs where both values were below the limit of detection were excluded. Horizontal red lines represent median values. *, *P* ≤ 0.05; **, *P* ≤ 0.01; ***, *P* ≤ 0.001; ****, *P* ≤ 0.0001. Download FIG S2, TIF file, 0.1 MB. Copyright © 2022 Sajadi et al. 2022 Sajadi et al. https://creativecommons.org/licenses/by/4.0/ This content is distributed under the terms of the Creative Commons Attribution 4.0 International license.

### SARS2-infected individuals had similar plasma neutralizing antibody levels regardless of severity of infection.

**FIG 4:**
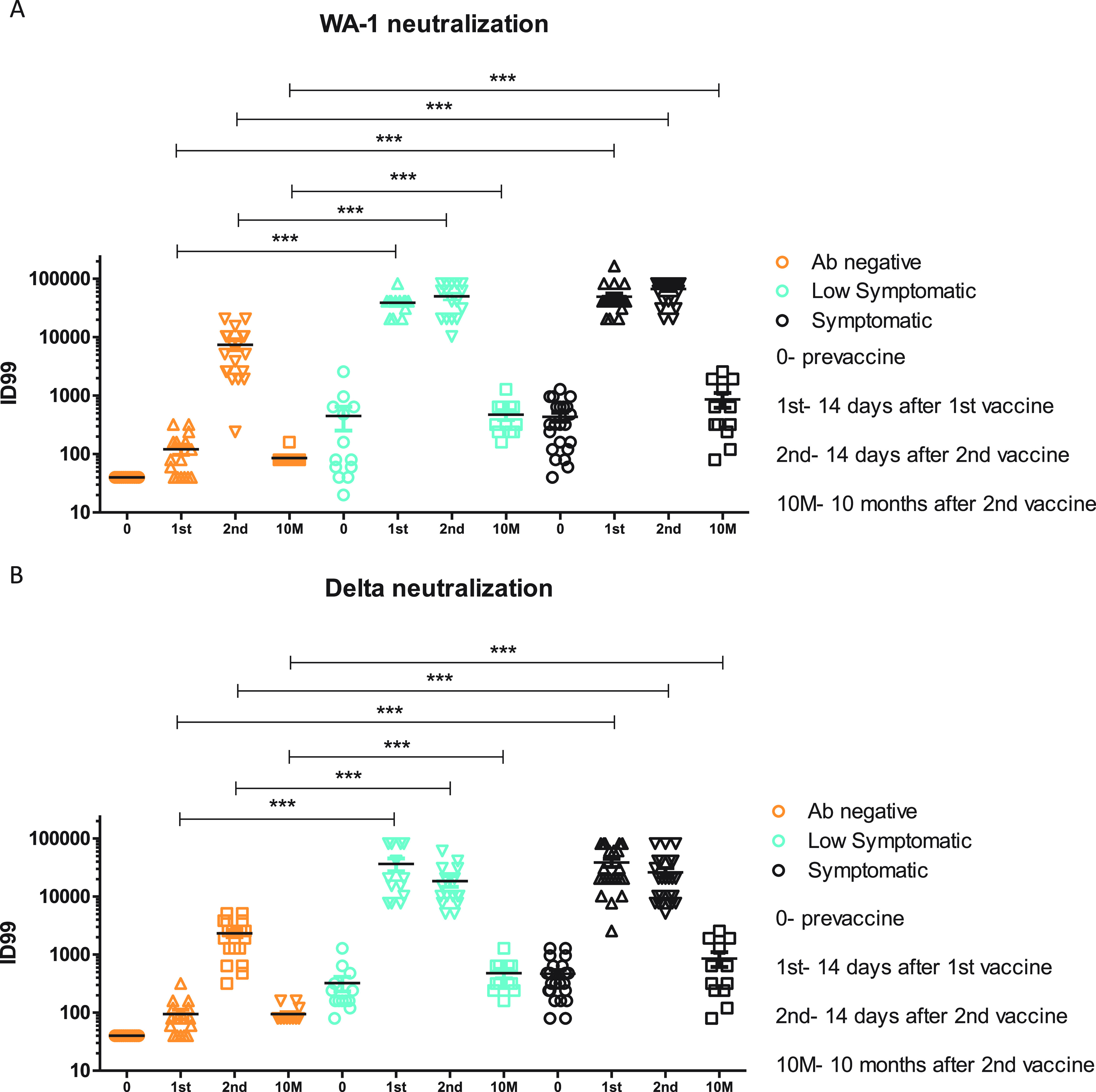
Live virus neutralization in plasma against WA-1 (A) and B.1.617.2 (Delta) (B) measured at baseline, at 14 days after 1st vaccination, and prior to and 14 days after the 2nd vaccination. ID_99_ is defined as highest dilution at which 99% cells were protected. Horizontal red lines represent median values. The lowest dilution tested was 1:40 (dotted black line). *, *P* ≤ 0.05; **, *P* ≤ 0.01; ***, *P* ≤ 0.001; ****, *P* ≤ 0.0001.

The plasma samples were tested against the WA-1 and B.1.617.2 (Delta) variants of SARS2. In the Ab-negative group, the neutralization titers for both Wuhan and Delta variants sequentially increased until 14 days after the second vaccination (Fig. 4A and B). In the low symptomatic and symptomatic groups, the endpoint titers peaked after the first vaccination and remained stable before and after the second vaccination. By 14 days after the 1st vaccination, median ID99 WA-1 neutralization titers were 80 in the Ab-negative group, compared to 40,960 in the low symptomatic group and 40,960 in the symptomatic group (*P* ≤ 0.001 for each) (Fig. 4A). Comparing Moderna versus Pfizer recipients within each group, we noted larger neutralization responses in SARS2-infected volunteers against WA-1 14 days after the first vaccine (*P* = 0.004) but not at any other time points (Fig. S1E and Table S1A).

### After the 1st vaccination, cellular responses were similar in previously SARS2-infected individuals.

**FIG 5:**
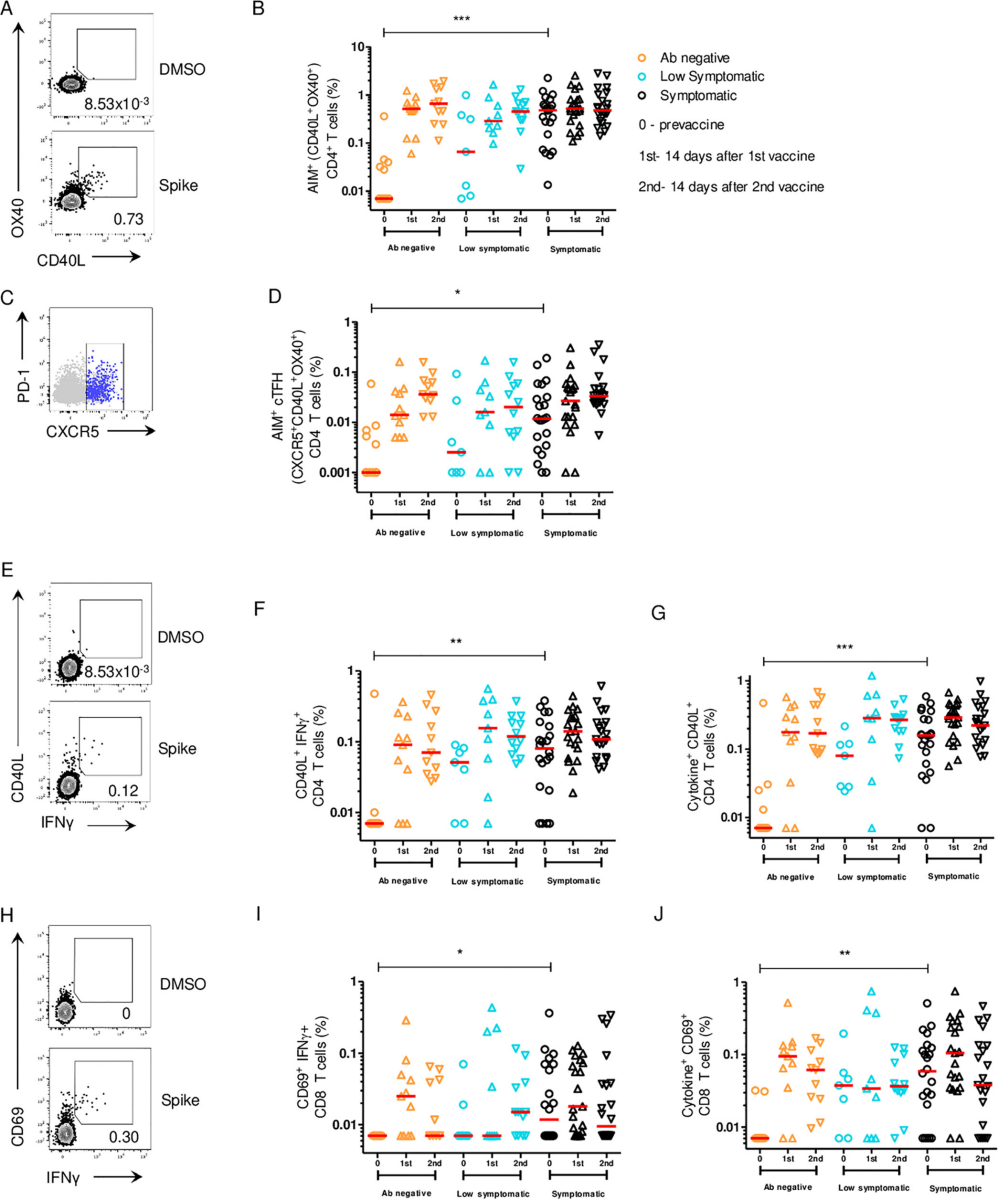
SARS2 S-specific CD4^+^ and CD8^+^ T cells following vaccination in unexposed and previously infected individuals. (A) Representative gating strategy of AIM^+^ (surface CD40L^+^ OX40^+^) CD4^+^ T cells stimulated with S peptide pool or left unstimulated (DMSO). (B) AIM^+^ S-specific CD4^+^ T cells of total CD4^+^ T cells. LOD, 0.007%. (C) Representative gating strategy of AIM^+^ (CD40L^+^ OX40^+^) circulating T follicular helper (cT_FH_) cells (blue, CXCR5^+^) overlaid on total CD4^+^ T cells (gray). (D) AIM^+^ S-specific cT_FH_ cells of total CD4^+^ T cells. LOD, 0.01%. (E) Representative gating strategy of CD40L^+^ IFN-γ^+^ CD4^+^ T cells. (F) Spike-specific CD40L^+^ IFN-γ^+^ CD4^+^ T cells of total CD4^+^ T cells. LOD, 0.007%. (G) Spike-specific CD40L^+^ CD4^+^ T cells producing IFN-γ, IL-2, or TNF-α of total CD4^+^ T cells. LOD, 0.007%. (H) Representative gating strategy of CD69^+^ IFN-γ^+^ CD8^+^ T cells. (I) Spike-specific CD69^+^ IFN-γ^+^ CD8^+^ T cells of total CD8^+^ T cells. LOD, 0.007%. (J) Spike-specific CD69^+^ CD8^+^ T cells producing IFN-γ, IL-2, or TNF-α of total CD8^+^ T cells. LOD, 0.007%. All data are background subtracted. Horizontal red lines represent median values. *, *P* ≤ 0.05; **, *P* ≤ 0.01; ***, *P* ≤ 0.001; ****, *P* ≤ 0.0001.

We compared the CD4^+^ and CD8^+^ T cell reactivities of the three cohorts against SARS2 S by activation-induced cell marker (AIM) and intracellular cytokine staining (ICS) assays (Fig. 5A, C, E and H and Fig. S3 and S4). For CD4 T cells, ICS can miss a large fraction of the virus-specific response, as CD4 T cells can have diverse functionalities; this is particularly true for T follicular helper (T_FH_) cells, with most antigen-specific GC-T_FH_ cells not making detectable amounts of any cytokines by ICS (8, 9). As a cytokine-agnostic approach, AIM assays often identify substantially more antigen-specific CD4 T cells than ICS (8–11). ICS serves as a complementary approach to identify CD4 T cells expressing specific cytokines of interest. When the AIM responses at baseline were compared, the symptomatic group was found to have significantly higher levels of CD4 and circulating T follicular helper (cT_FH_) responses to spike than the antibody-negative group (*P* ≤ 0.001 and *P* < 0.05, respectively) (Fig. 5B and D). Approximately 41% of SARS2-naive individuals had detectable spike-specific CD4^+^ T cells prior to vaccination. (Fig. 5B). The antibody-negative group saw increases in CD4 and cT_FH_ AIM responses after each vaccination, while AIM responses in the low symptomatic and symptomatic groups peaked after the first vaccination (Fig. 5B and D). There was no statistically significant difference between the AIM responses to CD4 or cT_FH_ between the SARS2-naive and infected groups after the 1st and 2nd vaccinations (Fig. 5B and D).

10.1128/msphere.00279-22.3 FIG S3 Spike-specific CD4^+^ and CD8^+^ T cells. (A) Gating strategy to define CD4^+^ and CD8^+^ T cells for AIM and ICS assays. (B) Representative gating strategy of CD40L^+^ IL-2^+^ and CD40L^+^ TNF-α^+^ CD4^+^ T cells stimulated with spike peptide pool or left unstimulated (DMSO). (C) Spike-specific CD40L^+^ TNF-α^+^ CD4^+^ T cells of total CD4^+^ T cells. (D) Spike-specific CD40L^+^ IL-2^+^ CD4^+^ T cells of total CD4^+^ T cells. (E) Representative gating strategy of CD69^+^ IL-2^+^ and CD69^+^ TNF-α^+^ CD8^+^ T cells. (F) Spike-specific CD69^+^ TNF-α^+^ CD8^+^ T cells of total CD8^+^ T cells. (G) Spike-specific CD69^+^ IL-2^+^ CD8^+^ T cells of total CD8^+^ T cells. All data are background subtracted. Horizontal red lines represent median values. Download FIG S3, TIF file, 0.1 MB. Copyright © 2022 Sajadi et al. 2022 Sajadi et al. https://creativecommons.org/licenses/by/4.0/ This content is distributed under the terms of the Creative Commons Attribution 4.0 International license.

10.1128/msphere.00279-22.4 FIG S4 Non-spike-specific CD4^+^ and CD8^+^ T cells. (A) Non-spike-specific AIM^+^ (surface CD40L^+^ OX40^+^) CD4^+^ T cells of total CD4^+^ T cells. (B) Non-spike-specific CD40L^+^ IFN-γ^+^ CD4^+^ T cells of total CD4^+^ T cells. (C) Non-spike-specific CD69^+^ IFN-γ^+^ CD8^+^ T cells of total CD8^+^ T cells following stimulation with MP_R. All data are background subtracted. Horizontal red lines represent median values. Download FIG S4, TIF file, 0.1 MB. Copyright © 2022 Sajadi et al. 2022 Sajadi et al. https://creativecommons.org/licenses/by/4.0/ This content is distributed under the terms of the Creative Commons Attribution 4.0 International license.

In the CD4^+^ ICS assay, significantly lower S-specific gamma interferon (IFN-γ) and cytokine staining were seen in the antibody-negative group at baseline than in the symptomatic groups, as expected (*P* ≤ 0.01 and *P* ≤ 0.001, respectively) (Fig. 5F and G). After the 1st vaccination, percentages of IFN-γ- and cytokine-secreting cells significantly increased in the antibody-negative group only (*P* = 0.02 and *P* = 0.02, respectively). Second vaccination did not see further cytokine increases in any of the groups (Fig. 5F and G).

S-specific CD8^+^ T cell IFN-γ and cytokine expression differences were noted between the symptomatic and antibody-negative groups at baseline (*P* < 0.05 and *P* ≤ 0.01, respectively) (Fig. 5I and J). After the 1st vaccination, percentages of IFN-γ- and cytokine-secreting cells significantly increased in the antibody-negative group only (*P* = 0.003 and *P* = 0.001, respectively). The percentages of IFN-γ- and cytokine-secreting cells in the low symptomatic and symptomatic groups did not significantly change before and after vaccinations (Fig. 5I and J). There was a strong positive correlation between the AIM^+^ CD4^+^ cells and percentage of IFN-γ-secreting CD4^+^ cells in each of the antibody-negative (*P* < 0.0001), low symptomatic (*P* = 0.003), and symptomatic (*P* < 0.0001) groups (Fig. S5). In the antibody-negative group, Moderna vaccine recipients had statistically significantly higher S-specific CD4^+^ T cells and cT_FH_ cells detected by AIM assay and higher S-specific CD4^+^ T cell and CD8^+^ T cell ICS responses than Pfizer recipients 14 days after the second vaccination (Fig. S6 and S7).

10.1128/msphere.00279-22.5 FIG S5 Correlation between spike-specific AIM^+^ and ICS^+^ CD4^+^ T cells. Shown is correlation between AIM^+^ (surface CD40L^+^ OX40^+^) and CD40L^+^ IFN-γ^+^ CD4^+^ T cells in antibody-negative (A) and SARS2-exposed (B and C) individuals. Statistics were calculated using Spearman’s correlation. Download FIG S5, TIF file, 0.1 MB. Copyright © 2022 Sajadi et al. 2022 Sajadi et al. https://creativecommons.org/licenses/by/4.0/ This content is distributed under the terms of the Creative Commons Attribution 4.0 International license.

10.1128/msphere.00279-22.6 FIG S6 Spike-specific CD4^+^ and cT_FH_ cells detected by AIM assay in Moderna and Pfizer recipients. AIM^+^ (surface CD40L^+^ OX40^+^) spike-specific CD4^+^ T cells and cT_FH_ cells of total CD4^+^ T cells in antibody-negative (orange) (A and E), low symptomatic (blue) (B and F), symptomatic (black) (C and G), and SARS2-infected (combined low symptomatic/symptomatic) (D and H) groups are shown. 1^st^, 14 days after first vaccination; 2^nd^, 14 days after second vaccination. Horizontal red lines represent medians. Download FIG S6, TIF file, 0.1 MB. Copyright © 2022 Sajadi et al. 2022 Sajadi et al. https://creativecommons.org/licenses/by/4.0/ This content is distributed under the terms of the Creative Commons Attribution 4.0 International license.

10.1128/msphere.00279-22.7 FIG S7 Spike-specific CD4^+^ and CD8^+^ cells detected by ICS assay in Moderna and Pfizer recipients. Spike-specific CD40L^+^ IFN-γ^+^ CD4^+^ T cells of total CD4^+^ T cells in antibody-negative (orange) (A and E), low symptomatic (blue) (B and F), symptomatic (black) (C and G), and SARS2-infected (combined low symptomatic/symptomatic) (D and H) groups are shown. Spike-specific CD69^+^ IFN-γ^+^ CD8^+^ T cells of total CD8^+^ T cells in antibody-negative (E), low symptomatic (F), symptomatic (G), and previously infected (low symptomatic/symptomatic) (H) groups are also shown. Horizontal red lines represent medians. Download FIG S7, TIF file, 0.1 MB. Copyright © 2022 Sajadi et al. 2022 Sajadi et al. https://creativecommons.org/licenses/by/4.0/ This content is distributed under the terms of the Creative Commons Attribution 4.0 International license.

The cellular and antibody data were analyzed for correlations. In the previously SARS2-infected groups, there were positive correlations between S-specific CD4^+^ cells (AIM^+^) at baseline with S IgG titers prevaccination (*P* = 0.01), at month 3 (*P* = 0.03) and at month 10 (*P* = 0.006) (Fig. S8A, I, and K). In the previously SARS2-infected groups, there were also positive correlations between AIM^+^ cT_FH_ cells at baseline with S IgG titers at month 10 (*P* = 0.04) (Fig. S8L), as well as AIM^+^ cT_FH_ cells at peak (14 days after 1st vaccination) with neutralization titers to WA-1 day 14 after the 1st vaccination (Table S1B). There were positive correlations between AIM^+^ CD4^+^ cells at baseline with neutralization titers to WA-1 prevaccination (*P* = 0.047) and day 14 after the second vaccination (*P* = 0.003) (Fig. S9A and E).

10.1128/msphere.00279-22.8 FIG S8 Correlation between preexisting spike-specific CD4^+^ T cells and spike IgG. Correlation between preexisting spike-specific AIM^+^ (surface CD40L^+^ OX40^+^) CD4^+^ T cells (A, C, E, G, I, and K) or AIM^+^ (surface CD40L^+^ OX40^+^) cT_FH_ cells (B, D, F, H, J, and K) and spike IgG prior to vaccination, 7 days after the 1st vaccination, 14 days after the 1st vaccination, 14 days after the 2nd vaccination, 3 months after the 2nd vaccination, and 10 months after the 2nd vaccination is shown. Low symptomatic and symptomatic groups are shown in blue and black circles, respectively. Statistics were calculated using Spearman’s correlation. Download FIG S8, TIF file, 0.1 MB. Copyright © 2022 Sajadi et al. 2022 Sajadi et al. https://creativecommons.org/licenses/by/4.0/ This content is distributed under the terms of the Creative Commons Attribution 4.0 International license.

10.1128/msphere.00279-22.9 FIG S9 Correlation between preexisting spike-specific CD4^+^ T cells and neutralization. Correlation between preexisting spike-specific AIM^+^ (surface CD40L^+^ OX40^+^) CD4^+^ T cells (A, C, E, and G) or AIM^+^ (surface CD40L^+^ OX40^+^) cT_FH_ cells (B, D, F, and H) and ID_99_ neutralization prior to vaccination, 14 days after the 1st vaccination, and 14 days after the 2nd vaccination. Low symptomatic and symptomatic groups are shown in blue and black circles, respectively. Statistics were calculated using Spearman’s correlation. Download FIG S9, TIF file, 0.1 MB. Copyright © 2022 Sajadi et al. 2022 Sajadi et al. https://creativecommons.org/licenses/by/4.0/ This content is distributed under the terms of the Creative Commons Attribution 4.0 International license.

## DISCUSSION

Several years into the COVID-19 pandemic, key questions about the immune response to SARS2 S protein after natural infection and vaccination remain. Currently, waning mRNA vaccine immunity has been demonstrated in a number of studies (3, 12–15), and thus, understanding the primary and secondary responses to SARS2 is imperative. In this study, we had the opportunity to evaluate primary responses in SARS2-naive vaccinees, as well as secondary responses in SARS2-infected vaccinees with differing severities of primary infection. The primary and secondary responses had a clear set of differences, including reactogenicity, antibody levels, and class of antibody (IgA), that were associated with severity of the primary infection.

After the first dose of vaccine, those with a history of SARS2 infection had more symptoms (reactogenicity), consistent with a secondary immune response. Interestingly, those in the symptomatic group also had a higher percentage of vaccine-related systemic symptoms than the low symptomatic group. The elevated reactogenicity to vaccination could be related to a general exaggerated response to antigens in this group (genetic differences) or a reflection of the immune response to a higher viral burden during SARS2 infection (a consequence of having more severe COVID-19) and somehow tied to the lower nasal/systemic IgA levels, though we could not find any evidence of this in past studies.

Prior to vaccination, the previously SARS2-infected groups had elevated levels of AIM^+^ CD4 and CD8 cells directed at the S protein of SARS2, cells that contributed to the secondary response after vaccination. However, there was also a high level of preexisting cross-reactive SARS2 CD4 T cells seen in the antibody-negative group, likely as a result of previous seasonal coronavirus infection, as seen in other studies (16–19). Within the previously SARS2-infected groups, those in the symptomatic group tended to have slightly higher baseline levels than the low symptomatic group. By 14 days after the first vaccination, the cellular responses in all 3 groups reached a peak, with no apparent changes at day 14 after the second vaccination.

The findings of higher binding and neutralization titers in vaccinees with prior SARS2 infection are indicative of a secondary immune response, and they have been noted in other studies as well (20–30). We have previously shown that peak titers in the symptomatic and low symptomatic groups occur by day 7 after the first vaccination (24), while peak titers in the Ab-negative group occur after the second vaccination. Two recent studies have documented protection from infection in vaccinees with prior SARS2 infection (1, 2); however, it remains unknown if 2 doses rather than 1 dose of an mRNA vaccine are needed for protection. Importantly, one of these studies has shown that SARS2-infected vaccinees appear to have less reinfection than SARS2-naive vaccinees (2), and the finding of elevated binding and neutralization titers in the former group could explain the increased protection, though cellular responses can contribute as well.

Among those with a history of SARS2 infection, there were differences noted in the primary response to infection, as well the secondary response to vaccination, that suggest that severity of infection dictates or skews IgA primary antibody responses, as well as memory responses in response to reexposure. It is known that severity of COVID-19 is tied to both elevated plasma IgA and IgG titers to SARS2 spike and receptor-binding domain (RBD) during acute infection (5, 6, 31, 32). Although we did not have samples from the acute infection in those with a history of SARS2 infection, prior to vaccination (and thus in the convalescence phase after primary infection), IgG titers in the plasma and nose were similar regardless of severity of past infection, while IgA titers were higher in the plasma and nose of those with less severe infection. This suggests that beyond the acute phase, there is an overall skewing of the response away from IgA in those with severe acute infection. Importantly, the skewing of the response away from IgA also continued after vaccination in those with more severe infection. Those with less severe infection had higher IgA levels in the plasma and nasal samples at nearly every time point measured, while IgG responses were equal in the two groups. This suggests that the severity of infection not only affected the primary antibody responses but also dictated the recall responses. Of note also was the trend toward the severe group having more reactogenicity. All of the above suggests that immune imprinting to the first SARS2 exposure dictates future responses. One other possibility is that the imprinting occurred prior to the first SARS2 exposure, to one of the seasonal coronaviruses, as some studies have shown. Regardless of the cause of the imprinting, it remains to be seen whether this will continue to newer strains and what effect reversing the order of SARS2 exposure (vaccination and then infection) will have on this.

This study was continued 10 months after the second vaccination, so there was an opportunity to study the early durability of the responses in the different groups. There was no difference in the slope of IgG decline between the groups, but again here a difference in the IgA slope was noted, with those with more severe infection having a steeper decline than those with less severe SARS2 infection. These data are consistent with the observation noted in this study of the severe group having lower convalescent-phase IgA levels despite a number of reports showing higher IgA levels during acute infection (5, 6, 31, 32). Interestingly, the AIM^+^ CD4^+^ cell response at baseline in the SARS2-infected correlated with IgG anti-S antibody responses, but only at baseline, month 3, and month 10. Given the lack of correlation with peak titers, this suggests that AIM^+^ CD4^+^ response tracks with the durability rather than the magnitude of the IgG response. Neutralizing antibody also correlated with AIM^+^ CD4^+^ cells at baseline and 14 days after vaccination, but it is unknown if neutralizing antibody correlations with AIM^+^ CD4^+^ cells also tracks with durability. Currently, there is a lack of data regarding the level of antibodies needed for protection, as well as the late vaccine efficacy (VE) in those with prior SARS2 infection, but data generated in this study can help the interpretation of VE and correlates of protection studies.

Mucosal immunity is an understudied but important facet of COVID-19 humoral immunity, as the respiratory mucosa is the first site of contact between SARS2 and humans. Recent studies have highlighted the presence of IgA and IgG in saliva after vaccination against SARS2 in humans (33–35). In saliva, IgA1 appears to be the predominant subtype raised against SARS2 vaccination (35). It is known that in the nasal mucosa, IgA and IgG are abundant, with IgA predominating (36), results that were seen in the non-vaccine-specific nasal responses in this study. Most of the IgA is thought to be secretory, in the form of dimers, produced locally and transported to mucosal surfaces via the polymeric immunoglobulin receptor (pIgR) (37). In contrast, IgG produced in the bone marrow or lymph nodes arrives by a process of transcytosis involving the FcRn receptor (38). Anti-S trimer IgA and IgG were not detected in all SARS2-infected volunteers at baseline, but this may be due to the fact that swabs were placed in 1 mL of phosphate-buffered saline (PBS) and thus diluted 1,000 times what their concentration would be on the mucosal surface prior to testing. Nevertheless, the S-specific IgG/IgA ratio in the SARS2-infected individuals prevaccination was much lower in the nose than in the blood, suggesting local IgA production. After vaccination, anti-S IgG titers in the nasal mucosa outstripped IgA, as reflected in the skewed IgG/IgA ratios, some of which exceeded 100:1. These changes mirrored the concentrations measured in plasma, whereby IgG levels outpaced IgA, which is logical given the route of vaccination (intramuscular [i.m.]). However, despite lower comparative levels, IgA (systemic and mucosal) could still have a key role in protection. Unfortunately, there was not enough sample to carry out functional studies on the nasal samples.

The presence of both IgG and IgA at the site of first viral contact is important in that they are likely involved in the process by which the vaccines afford protection from acquisition and subsequent disease. Although plasma IgG binding and neutralizing antibody have been the focus of correlative studies (39, 40), more recent studies have focused on the potential role of IgA. One recent study has shown that anti-SARS2 S dimeric IgA (which would be present in the nasal mucosa) is about 15 times more potent than monomeric IgA (41). Two recent studies have shown that serum IgA in response to vaccination and natural infection is associated with protection from SARS2 infection (42, 43). The findings in the current study are important, because if the contribution of IgA proves to be more important than that of IgG for protection against reinfection, those with a history of more severe disease may be at elevated risk of reinfection. As noted above, our data suggest that vaccination cannot overcome the apparent imprinting based on severity of initial infection.

As more of the population becomes infected after vaccination, the question also arises how the sequence of events (vaccination and infection) and treatment (antivirals, monoclonal antibodies, and immune modulators) can also affect the immune response. The relative importance of each immunoglobulin class could differ based on whether an individual was infected or vaccinated, as local IgA production after infection may be a significant contributor to mucosal immunity compared to vaccination, in which most of the antibodies arrive from the systemic circulation. Interestingly, while we did measure antibody attached to secretory protein in the nasal samples of those vaccinated (presumably IgA because IgM was undetectable in all but a few samples), further studies need to be carried out to definitively identify the type and source of these antibodies.

The two vaccines used in this study, Moderna mRNA-1273 and Pfizer BNT162b2, share similarities in design and mode of action; however, they differ in the dosage administered, with the Moderna vaccine using 3.3 times more (100 μg versus 30 μg). We noted differences in cellular responses in the SARS2-naive group 2 weeks after the second vaccination in all CD4, cT_FH_, and CD8 assays that we analyzed, with the Moderna group having elevated responses. In contrast, in the SARS2-infected group, fewer differences were noted, with only cT_FH_ responses noted to be elevated in the Moderna recipients in the symptomatic group 2 weeks after first vaccination.

The strengths of this study include using a clearly defined cohort with regimented sampling points. Additional strengths include use of vaccinees who received Moderna or Pfizer, as well as studying clinical, cellular, and mucosal parameters. This study was limited by sample size, and thus, a larger sample size could have found additional differences between the groups based on prior SARS2 exposure, type of vaccination, or other factors. Finally, although the studies undertaken were fairly comprehensive, some key sites involved in immune regulation (lymph nodes and lungs) were not sampled in the current study, and functional studies of antibodies in the mucosal compartment were not carried out (due to sample limitations).

This study found key differences in the response to SARS2 vaccination depending on previous exposure and severity of primary infection. In the previously SARS2-infected subjects, severe infection appeared to skew the response away from IgA, decreasing systemic and mucosal IgA, effects that linger after subsequent vaccination and which could be important in protection from reinfection. SARS2-infected individuals had higher binding and neutralization titers throughout the study. This was also true of the relevant classes of anti-S antibody (IgG and IgA) at mucosal sites, which are likely involved in the protective efficacy of the mRNA vaccines. Finally, while differences in cellular immunity were less evident between groups after vaccination, there were data supporting the idea that the AIM^+^ CD4^+^ cell response could be tied to durability of humoral immunity against the SARS2 S protein. However, the lingering differences between the SARS2-infected and SARS2-naive subjects up to 10 months postvaccination suggest that additional strategies (such as boosting of the SARS2-naive vaccinees) are needed to narrow the immunological differences observed between the groups in this study, which are likely related to the number of exposures to the SARS2 envelope trimer. Further studies will be necessary to determine whether these differences translate into higher risk of reinfection.

## MATERIALS AND METHODS

### Study design.

HCW who had previously enrolled in a hospital-wide serosurvey study in the summer of 2020 (7), conducted at the University of Maryland Medical Center, were randomly contacted. These HCW had all received antibody tests to categorize them into antibody negative and antibody positive. The antibody-positive group was then asked about their symptoms with the initial infection. This study then followed three stratified groups as previously described (24): SARS2 IgG antibody negative (Ab negative), IgG positive asymptomatic and minimally symptomatic (asymptomatic or ≤2 days of missed work on initial infection) COVID-19 (low symptomatic); and IgG positive with history of symptomatic (≥3 days of missed work on initial infection with or without hospitalization) COVID-19 (symptomatic). Work days missed were based on severity of symptoms and not mandatory quarantine. Participants were vaccinated with either the Pfizer-BioNTech or Moderna vaccine, depending on personal preference and availability. Blood was drawn at day 0 (or baseline), days 7, 10, and 14 after the first vaccination, and days 0, 7, 10, 14, and 28, as well as months 3 and 10, after the second vaccination. Plasma and peripheral blood mononuclear cells (PBMCs) were immediately separated and frozen prior to being used. Upon study enrollment, volunteers were given a questionnaire regarding COVID-19 history, and after vaccination, they were given a questionnaire regarding postvaccination symptoms (reactogenicity). This included presence of subjective fever, chills, fatigue, myalgia, headache, arthralgia, axillary lymphadenopathy, injection site pain, injection site swelling, injection site erythema, and nausea/vomiting.

### ELISA.

IgM, IgA, and IgG responses were measured in the plasma and nasal samples. Additionally, antibody responses to the secretory component (SC), indicative of secretory IgA or IgM, were evaluated in nasal and plasma samples. For nasal samples, the anterior nares were swabbed with a flock swab that was wetted with PBS prior to swabbing, and then the swab was placed in a tube with 1 mL of PBS. IgA, IgM, IgG, and secretory component (SC) enzyme-linked immunosorbent assays (ELISAs) to detect SARS2 S trimer were made by modifying an assay (44) to give a readout of endpoint binding titers. Modifications include the lowest dilution of antibody tested (for plasma samples, 1:20 for IgM, 1:100 for IgA, and 1:50 for IgG; for nasal samples, 1:1,000 for IgM, IgA, and IgG), as well as the secondary antibody used (for plasma samples, 1:500 for IgM, 1:1,000 for IgA, and 1:4,000 for IgG; for nasal samples, 1:500 for IgM, 1:1,000 for IgA, and 1:500 for IgG).

### Neutralization assay.

Live virus neutralization assays were carried out, as previously described (24, 45), on plasma from days 0 and 14 after the first vaccination and day 14 after the second vaccination. Briefly, serial dilutions of plasma were incubated for 1 h with 200 median tissue culture infectious doses (TCID_50_) of SARS2 WA-1 (courtesy of Natalie Thornburg) or B.1.617.2 (hCoV-19/USA/MD-HP05285/2021, courtesy of Andrew Pekosz). This admixture was added to Vero E6 cells, and cytopathic effect was assayed visually. The 99% infectious dose (ID_99_) was recorded as the first plasma dilution with cytopathic effect similar to the virus-only control wells.

### AIM assay.

To quantify the S-specific T cell response, an S megapool (MP) containing overlapping peptides of the entire S protein sequence was used. In addition, MP remainder (MP_R) containing non-S epitopes in SARS2 was used, as previously described (16). PBMCs were incubated with 1 μg/mL of S MP or MP_R for 24 h in a 96-well U-bottom plate at 1 × 10^6^ cells/well in RPMI medium containing 5% human serum AB (Gemini Bio), 1% GlutaMAX (Thermo Fisher Scientific) and 1% penicillin-streptomycin (100 units/mL of penicillin and 100 μg/mL of streptomycin) (Thermo Fisher Scientific). Dimethyl sulfoxide (DMSO; 0.1%) and staphylococcal enterotoxin B (SEB; 1 μg/mL) (Toxin Technologies Inc.) were used as negative and positive controls, respectively. Fifteen minutes prior to the addition of the MPs, 0.5 μg/mL of anti-CD40 monoclonal antibody (MAb) (Miltenyi Biotec) and anti-CXCR5 (Fisher Scientific; 25-9185-42) were added. Following 24 h of incubation, PBMCs were surface stained with anti-CD14 (Biolegend; 301820), anti-CD16 (Biolegend; 302018), anti-CD20 (Biolegend; 302314), anti-CD3 (BD Biosciences; 552852), anti-CD4 (BD Biosciences; 560768), anti-CD8 (Biolegend; 301042), anti-PD-1 (Biolegend; 329930), anti-CD69 (Biolegend; 310922), anti-OX40 (Biolegend; 350008), anti-4-1BB (Biolegend; 309820), anti-CD40L (Biolegend; 310815), and fixable viability dye (Fisher Scientific; 65-0865-14) for 30 min at 4°C, washed in PBS, fixed for 10 min in 4% formaldehyde (Cytofix; BD Biosciences), washed in PBS, and acquired on a BD FACSCelesta cell analyzer. All antibodies were used at a 1:200 dilution unless otherwise noted. Fixable viability dye was used at a 1:1,000 dilution. Activation-induced cell marker-positive (AIM^+^) antigen-specific CD4^+^ T cells were defined as surface CD40L^+^ OX40^+^, and antigen-specific circulating T follicular helper (cT_FH_) cells were defined as CXCR5^+^ surface CD40L^+^ OX40^+^.

### Intracellular cytokine staining (ICS) assay.

PBMCs were incubated with 1 μg/mL of S MP or MP_R for 24 h in a 96-well U-bottom plate at 1 × 10^6^ cells/well in RPMI medium containing 5% human serum AB (Gemini Bio), 1% GlutaMAX (Thermo Fisher Scientific), and 1% penicillin-streptomycin (Thermo Fisher Scientific), as previously described (46). DMSO (0.1%) and 1 μg/mL of SEB were used as negative and positive controls, respectively. Fifteen minutes prior to the addition of the MPs, 0.5 μg/mL of anti-CD40 MAb (Miltenyi Biotec) and anti-CXCR5 (Fisher Scientific, 25-9185-42) were added. Following 24 h of incubation, monensin (GolgiStop; BD Biosciences) and brefeldin A (GolgiPlug; BD Biosciences) were added and PBMCs were incubated for an additional 4 h. PBMCs were surface stained with anti-CD14 (Biolegend; 301820), anti-CD16 (Biolegend; 302018), anti-CD20 (Biolegend; 302314), anti-CD3 (BD Biosciences; 552852), anti-CD4 (BD Biosciences; 560768), anti-CD8 (Biolegend; 301042), anti-PD-1 (Biolegend; 329930), anti-CD69 (Biolegend; 310922), anti-4-1BB (Biolegend; 309822), anti-CD40L (Biolegend; 310806) and fixable viability dye (Fisher Scientific; 65-0865-14) for 30 min at 4°C, washed in PBS, and then fixed and permeabilized (1× Cytofix/Cytoperm; BD Biosciences) for 30 min. After fixation, PBMCs were washed in 1× Perm/Wash buffer (BD Biosciences) and stained for 45 min at 4°C with anti-interleukin-2 (anti-IL-2; BD Biosciences; 554567), anti-IFN-γ (Biolegend; 502515), and anti-tumor necrosis factor alpha (anti-TNF-α; Biolegend; 502920) and acquired on a BD FACSCelesta cell analyzer. All antibodies were used at a 1:200 dilution unless otherwise noted. Fixable viability dye was used at a 1:1,000 dilution. Antigen-specific CD4^+^ T cells were defined as CD40L^+^ and IFN-γ^+^, TNF-α^+^, or IL-2^+^, and antigen-specific CD8^+^ T cells were defined as CD69^+^ and IFN-γ^+^, TNF-α^+^, or IL-2^+^.

### Statistical analysis.

The reciprocal endpoint binding titers represent the maximal dilution of plasma that achieves binding. For T cell assays, sample quality was assessed by CD4^+^ AIM^+^ (CD40L^+^ OX40^+^) SEB response. Samples with an SEB CD4^+^ AIM^+^ response lower than one-half the median (<3.7% CD4^+^ AIM^+^ of total CD4^+^ T cells) were eliminated from the analysis (in this study, out of all 137 samples run for the AIM/ICS assays, 8 were eliminated [5%]). T cell data were analyzed using FlowJo 10.7.1. All data were background subtracted. Statistical analysis was carried out with GraphPad Prism 5 (GraphPad Software). Outlier analysis was not performed. The study groups were compared separately at each time point using one-way analysis of variance (ANOVA) (Kruskal-Wallis test with Dunn’s multiple-comparison test as the posttest). Pairwise differences between groups for binary outcomes were assessed by the 2-tailed Fisher’s exact test. Pairwise differences between groups for quantitative outcomes were assessed by Mann-Whitney tests, with a *P* value of <0.05 being considered significant. Correlation graphs were analyzed using Spearman’s correlation test. All volunteers in this study provided informed consent for the institutional review board (IRB)-approved study (University of Maryland, Baltimore, MD).

## References

[1] Cavanaugh AM, Spicer KB, Thoroughman D, Glick C, Winter K. 2021. Reduced risk of reinfection with SARS-CoV-2 after COVID-19 vaccination—Kentucky, May–June 2021. MMWR Morb Mortal Wkly Rep 70:1081–1083. doi:10.15585/mmwr.mm7032e1. 34383732 PMC8360277

[2] Pouwels KB, Pritchard E, Matthews PC, Stoesser N, Eyre DW, Vihta K-D, House T, Hay J, Bell JI, Newton JN, Farrar J, Crook D, Cook D, Rourke E, Studley R, Peto TEA, Diamond I, Walker AS. 2021. Effect of Delta variant on viral burden and vaccine effectiveness against new SARS-CoV-2 infections in the UK. Nat Med 27:2127–2135. doi:10.1038/s41591-021-01548-7. 34650248 PMC8674129

[3] Thomas SJ, Moreira ED, Kitchin N, Absalon J, Gurtman A, Lockhart S, Perez JL, Pérez Marc G, Polack FP, Zerbini C, Bailey R, Swanson KA, Xu X, Roychoudhury S, Koury K, Bouguermouh S, Kalina WV, Cooper D, Frenck RW, Hammitt LL, Türeci Ö, Nell H, Schaefer A, Ünal S, Yang Q, Liberator P, Tresnan DB, Mather S, Dormitzer PR, Şahin U, Gruber WC, Jansen KU, C4591001 Clinical Trial Group. 2021. Safety and efficacy of the BNT162b2 mRNA Covid-19 vaccine through 6 months. N Engl J Med 385:1761–1773. doi:10.1056/NEJMoa2110345. 34525277 PMC8461570

[4] Hashem AM, Algaissi A, Almahboub SA, Alfaleh MA, Abujamel TS, Alamri SS, Alluhaybi KA, Hobani HI, AlHarbi RH, Alsulaiman RM, ElAssouli M-Z, Hala S, Alharbi NK, Alhabbab RY, AlSaieedi AA, Abdulaal WH, Bukhari A, AL-Somali AA, Alofi FS, Khogeer AA, Pain A, Alkayyal AA, Almontashiri NAM, Ahmad BM, Li X. 2020. Early humoral response correlates with disease severity and outcomes in COVID-19 patients. Viruses 12:1390. doi:10.3390/v12121390. 33291713 PMC7761967

[5] Benner SE, Patel EU, Laeyendecker O, Pekosz A, Littlefield K, Eby Y, Fernandez RE, Miller J, Kirby CS, Keruly M, Klock E, Baker OR, Schmidt HA, Shrestha R, Burgess I, Bonny TS, Clarke W, Caturegli P, Sullivan D, Shoham S, Quinn TC, Bloch EM, Casadevall A, Tobian AAR, Redd AD. 2020. SARS-CoV-2 antibody avidity responses in COVID-19 patients and convalescent plasma donors. J Infect Dis 222:1974–1984. doi:10.1093/infdis/jiaa581. 32910175 PMC7499592

[6] Klein SL, Pekosz A, Park H-S, Ursin RL, Shapiro JR, Benner SE, Littlefield K, Kumar S, Naik HM, Betenbaugh MJ, Shrestha R, Wu AA, Hughes RM, Burgess I, Caturegli P, Laeyendecker O, Quinn TC, Sullivan D, Shoham S, Redd AD, Bloch EM, Casadevall A, Tobian AA. 2020. Sex, age, and hospitalization drive antibody responses in a COVID-19 convalescent plasma donor population. J Clin Invest 130:6141–6150. doi:10.1172/JCI142004. 32764200 PMC7598041

[7] Mullins KE, Merrill V, Ward M, King B, Rock P, Caswell M, Ahlman M, Harris AD, Christenson R. 2021. Validation of COVID-19 serologic tests and large scale screening of asymptomatic healthcare workers. Clin Biochem 90:23–27. doi:10.1016/j.clinbiochem.2021.01.004. 33472036 PMC7813506

[8] Dan JM, Lindestam Arlehamn CS, Weiskopf D, da Silva Antunes R, Havenar-Daughton C, Reiss SM, Brigger M, Bothwell M, Sette A, Crotty S. 2016. A cytokine-independent approach to identify antigen-specific human germinal center T follicular helper cells and rare antigen-specific CD4+ T cells in blood. J Immunol 197:983–993. doi:10.4049/jimmunol.1600318. 27342848 PMC4955771

[9] Havenar-Daughton C, Reiss SM, Carnathan DG, Wu JE, Kendric K, Torrents de la Peña A, Kasturi SP, Dan JM, Bothwell M, Sanders RW, Pulendran B, Silvestri G, Crotty S. 2016. Cytokine-independent detection of antigen-specific germinal center T follicular helper cells in immunized nonhuman primates using a live cell activation-induced marker technique. J Immunol 197:994–1002. doi:10.4049/jimmunol.1600320. 27335502 PMC4955744

[10] Pauthner MG, Nkolola JP, Havenar-Daughton C, Murrell B, Reiss SM, Bastidas R, Prévost J, Nedellec R, von Bredow B, Abbink P, Cottrell CA, Kulp DW, Tokatlian T, Nogal B, Bianchi M, Li H, Lee JH, Butera ST, Evans DT, Hangartner L, Finzi A, Wilson IA, Wyatt RT, Irvine DJ, Schief WR, Ward AB, Sanders RW, Crotty S, Shaw GM, Barouch DH, Burton DR. 2019. Vaccine-induced protection from homologous tier 2 SHIV challenge in nonhuman primates depends on serum-neutralizing antibody titers. Immunity 50:241–252.e246. doi:10.1016/j.immuni.2018.11.011. 30552025 PMC6335502

[11] Zhang Z, Mateus J, Coelho CH, Dan JM, Moderbacher CR, Gálvez RI, Cortes FH, Grifoni A, Tarke A, Chang J, Escarrega EA, Kim C, Goodwin B, Bloom NI, Frazier A, Weiskopf D, Sette A, Crotty S. 2022. Humoral and cellular immune memory to four COVID-19 vaccines. Cell 185:2434–2451.E17. doi:10.1016/j.cell.2022.05.022. 35764089 PMC9135677

[12] Levin EG, Lustig Y, Cohen C, Fluss R, Indenbaum V, Amit S, Doolman R, Asraf K, Mendelson E, Ziv A, Rubin C, Freedman L, Kreiss Y, Regev-Yochay G. 2021. Waning immune humoral response to BNT162b2 Covid-19 vaccine over 6 months. N Engl J Med 385:e84. doi:10.1056/NEJMoa2114583. 34614326 PMC8522797

[13] Goldberg Y, Mandel M, Bar-On YM, Bodenheimer O, Freedman L, Haas EJ, Milo R, Alroy-Preis S, Ash N, Huppert A. 2021. Waning immunity after the BNT162b2 vaccine in Israel. N Engl J Med 385:e85. doi:10.1056/NEJMoa2114228. 34706170 PMC8609604

[14] Nanduri S, Pilishvili T, Derado G, Soe MM, Dollard P, Wu H, Li Q, Bagchi S, Dubendris H, Link-Gelles R, Jernigan JA, Budnitz D, Bell J, Benin A, Shang N, Edwards JR, Verani JR, Schrag SJ. 2021. Effectiveness of Pfizer-BioNTech and Moderna vaccines in preventing SARS-CoV-2 infection among nursing home residents before and during widespread circulation of the SARS-CoV-2 B.1.617.2 (Delta) variant—National Healthcare Safety Network, March 1–August 1, 2021. MMWR Morb Mortal Wkly Rep 70:1163–1166. doi:10.15585/mmwr.mm7034e3. 34437519 PMC8389386

[15] Tartof SY, Slezak JM, Fischer H, Hong V, Ackerson BK, Ranasinghe ON, Frankland TB, Ogun OA, Zamparo JM, Gray S, Valluri SR, Pan K, Angulo FJ, Jodar L, McLaughlin JM. 2021. Effectiveness of mRNA BNT162b2 COVID-19 vaccine up to 6 months in a large integrated health system in the USA: a retrospective cohort study. Lancet 398:1407–1416. doi:10.1016/S0140-6736(21)02183-8. 34619098 PMC8489881

[16] Grifoni A, Weiskopf D, Ramirez SI, Mateus J, Dan JM, Moderbacher CR, Rawlings SA, Sutherland A, Premkumar L, Jadi RS, Marrama D, de Silva AM, Frazier A, Carlin AF, Greenbaum JA, Peters B, Krammer F, Smith DM, Crotty S, Sette A. 2020. Targets of T cell responses to SARS-CoV-2 coronavirus in humans with COVID-19 disease and unexposed individuals. Cell 181:1489–1501.E15. doi:10.1016/j.cell.2020.05.015. 32473127 PMC7237901

[17] Mateus J, Grifoni A, Tarke A, Sidney J, Ramirez SI, Dan JM, Burger ZC, Rawlings SA, Smith DM, Phillips E, Mallal S, Lammers M, Rubiro P, Quiambao L, Sutherland A, Yu ED, da Silva Antunes R, Greenbaum J, Frazier A, Markmann AJ, Premkumar L, de Silva A, Peters B, Crotty S, Sette A, Weiskopf D. 2020. Selective and cross-reactive SARS-CoV-2 T cell epitopes in unexposed humans. Science 370:89–94. doi:10.1126/science.abd3871. 32753554 PMC7574914

[18] Swadling L, Diniz MO, Schmidt NM, Amin OE, Chandran A, Shaw E, Pade C, Gibbons JM, Le Bert N, Tan AT, Jeffery-Smith A, Tan CCS, Tham CYL, Kucykowicz S, Aidoo-Micah G, Rosenheim J, Davies J, Johnson M, Jensen MP, Joy G, McCoy LE, Valdes AM, Chain BM, Goldblatt D, Altmann DM, Boyton RJ, Manisty C, Treibel TA, Moon JC, van Dorp L, Balloux F, McKnight Á, Noursadeghi M, Bertoletti A, Maini MK, COVIDsortium Investigators. 2022. Pre-existing polymerase-specific T cells expand in abortive seronegative SARS-CoV-2. Nature 601:110–117. doi:10.1038/s41586-021-04186-8. 34758478 PMC8732273

[19] Loyal L, Braun J, Henze L, Kruse B, Dingeldey M, Reimer U, Kern F, Schwarz T, Mangold M, Unger C, Dörfler F, Kadler S, Rosowski J, Gürcan K, Uyar-Aydin Z, Frentsch M, Kurth F, Schnatbaum K, Eckey M, Hippenstiel S, Hocke A, Müller MA, Sawitzki B, Miltenyi S, Paul F, Mall MA, Wenschuh H, Voigt S, Drosten C, Lauster R, Lachman N, Sander L-E, Corman VM, Röhmel J, Meyer-Arndt L, Thiel A, Giesecke-Thiel C. 2021. Cross-reactive CD4(+) T cells enhance SARS-CoV-2 immune responses upon infection and vaccination. Science 374:eabh1823. doi:10.1126/science.abh1823. 34465633 PMC10026850

[20] Krammer F, Srivastava K, Alshammary H, Amoako AA, Awawda MH, Beach KF, Bermúdez-González MC, Bielak DA, Carreño JM, Chernet RL, Eaker LQ, Ferreri ED, Floda DL, Gleason CR, Hamburger JZ, Jiang K, Kleiner G, Jurczyszak D, Matthews JC, Mendez WA, Nabeel I, Mulder LCF, Raskin AJ, Russo KT, Salimbangon A-BT, Saksena M, Shin AS, Singh G, Sominsky LA, Stadlbauer D, Wajnberg A, Simon V. 2021. Antibody responses in seropositive persons after a single dose of SARS-CoV-2 mRNA vaccine. N Engl J Med 384:1372–1374. doi:10.1056/NEJMc2101667. 33691060 PMC8008743

[21] Ebinger JE, Fert-Bober J, Printsev I, Wu M, Sun N, Prostko JC, Frias EC, Stewart JL, Van Eyk JE, Braun JG, Cheng S, Sobhani K. 2021. Antibody responses to the BNT162b2 mRNA vaccine in individuals previously infected with SARS-CoV-2. Nat Med 27:981–984. doi:10.1038/s41591-021-01325-6. 33795870 PMC8205849

[22] Abu Jabal K, Ben-Amram H, Beiruti K, Batheesh Y, Sussan C, Zarka S, Edelstein M. 2021. Impact of age, ethnicity, sex and prior infection status on immunogenicity following a single dose of the BNT162b2 mRNA COVID-19 vaccine: real-world evidence from healthcare workers, Israel, December 2020 to January 2021. Euro Surveill 26:2100096. doi:10.2807/1560-7917.ES.2021.26.6.2100096. 33573712 PMC7879501

[23] Levi R, Azzolini E, Pozzi C, Ubaldi L, Lagioia M, Mantovani A, Rescigno M. 2021. One dose of SARS-CoV-2 vaccine exponentially increases antibodies in individuals who have recovered from symptomatic COVID-19. J Clin Invest 131:e149154. doi:10.1172/JCI149154. 33956667 PMC8203458

[24] Saadat S, Rikhtegaran Tehrani Z, Logue J, Newman M, Frieman MB, Harris AD, Sajadi MM. 2021. Binding and neutralization antibody titers after a single vaccine dose in health care workers previously infected with SARS-CoV-2. JAMA 325:1467–1469. doi:10.1001/jama.2021.3341. 33646292 PMC7922233

[25] Fraley E, LeMaster C, Geanes E, Banerjee D, Khanal S, Grundberg E, Selvarangan R, Bradley T. 2021. Humoral immune responses during SARS-CoV-2 mRNA vaccine administration in seropositive and seronegative individuals. BMC Med 19:169. doi:10.1186/s12916-021-02055-9. 34304742 PMC8310732

[26] Stamatatos L, Czartoski J, Wan Y-H, Homad LJ, Rubin V, Glantz H, Neradilek M, Seydoux E, Jennewein MF, MacCamy AJ, Feng J, Mize G, De Rosa SC, Finzi A, Lemos MP, Cohen KW, Moodie Z, McElrath MJ, McGuire AT. 2021. mRNA vaccination boosts cross-variant neutralizing antibodies elicited by SARS-CoV-2 infection. Science 372:1413–1418. doi:10.1126/science.abg9175. 33766944 PMC8139425

[27] Andreano E, Paciello I, Piccini G, Manganaro N, Pileri P, Hyseni I, Leonardi M, Pantano E, Abbiento V, Benincasa L, Giglioli G, De Santi C, Fabbiani M, Rancan I, Tumbarello M, Montagnani F, Sala C, Montomoli E, Rappuoli R. 2021. Hybrid immunity improves B cells and antibodies against SARS-CoV-2 variants. Nature 600:530–535. doi:10.1038/s41586-021-04117-7. 34670266 PMC8674140

[28] Wang Z, Muecksch F, Schaefer-Babajew D, Finkin S, Viant C, Gaebler C, Hoffmann Hans-H, Barnes CO, Cipolla M, Ramos V, Oliveira TY, Cho A, Schmidt F, Da Silva J, Bednarski E, Aguado L, Yee J, Daga M, Turroja M, Millard KG, Jankovic M, Gazumyan A, Zhao Z, Rice CM, Bieniasz PD, Caskey M, Hatziioannou T, Nussenzweig MC. 2021. Naturally enhanced neutralizing breadth against SARS-CoV-2 one year after infection. Nature 595:426–431. doi:10.1038/s41586-021-03696-9. 34126625 PMC8277577

[29] Goel RR, Apostolidis SA, Painter MM, Mathew D, Pattekar A, Kuthuru O, Gouma S, Hicks P, Meng W, Rosenfeld AM, Dysinger S, Lundgreen KA, Kuri-Cervantes L, Adamski S, Hicks A, Korte S, Oldridge DA, Baxter AE, Giles JR, Weirick ME, McAllister CM, Dougherty J, Long S, D’Andrea K, Hamilton JT, Betts MR, Luning Prak ET, Bates P, Hensley SE, Greenplate AR, Wherry EJ. 2021. Distinct antibody and memory B cell responses in SARS-CoV-2 naïve and recovered individuals following mRNA vaccination. Sci Immunol 6:eabi6950. doi:10.1126/sciimmunol.abi6950. 33858945 PMC8158969

[30] Manisty C, Otter AD, Treibel TA, McKnight Á, Altmann DM, Brooks T, Noursadeghi M, Boyton RJ, Semper A, Moon JC. 2021. Antibody response to first BNT162b2 dose in previously SARS-CoV-2-infected individuals. Lancet 397:1057–1058. doi:10.1016/S0140-6736(21)00501-8. 33640038 PMC7972310

[31] Yates JL, Ehrbar DJ, Hunt DT, Girardin RC, Dupuis AP, Payne AF, Sowizral M, Varney S, Kulas KE, Demarest VL, Howard KM, Carson K, Hales M, Ejemel M, Li Q, Wang Y, Peredo-Wende R, Ramani A, Singh G, Strle K, Mantis NJ, McDonough KA, Lee WT. 2021. Serological analysis reveals an imbalanced IgG subclass composition associated with COVID-19 disease severity. Cell Rep Med 2:100329. doi:10.1016/j.xcrm.2021.100329. 34151306 PMC8205277

[32] Semmler G, Traugott MT, Graninger M, Hoepler W, Seitz T, Kelani H, Karolyi M, Pawelka E, Aragón de La Cruz S, Puchhammer-Stöckl E, Aberle SW, Stiasny K, Zoufaly A, Aberle JH, Weseslindtner L. 2021. Assessment of S1-, S2-, and NCP-specific IgM, IgA, and IgG antibody kinetics in acute SARS-CoV-2 infection by a microarray and twelve other immunoassays. J Clin Microbiol 59:e02890-20. doi:10.1128/JCM.02890-20. 33602698 PMC8091850

[33] Azzi L, Dalla Gasperina D, Veronesi G, Shallak M, Ietto G, Iovino D, Baj A, Gianfagna F, Maurino V, Focosi D, Maggi F, Ferrario MM, Dentali F, Carcano G, Tagliabue A, Maffioli LS, Accolla RS, Forlani G. 2022. Mucosal immune response in BNT162b2 COVID-19 vaccine recipients. EBioMedicine 75:103788. doi:10.1016/j.ebiom.2021.103788. 34954658 PMC8718969

[34] Mades A, Chellamathu P, Kojima N, Lopez L, MacMullan MA, Denny N, Angel AN, Santacruz M, Casian JG, Brobeck M, Nirema N, Klausner JD, Turner F, Slepnev VI, Ibrayeva A. 2021. Detection of persistent SARS-CoV-2 IgG antibodies in oral mucosal fluid and upper respiratory tract specimens following COVID-19 mRNA vaccination. Sci Rep 11:24448. doi:10.1038/s41598-021-03931-3. 34961780 PMC8712521

[35] Darwich A, Pozzi C, Fornasa G, Lizier M, Azzolini E, Spadoni I, Carli F, Voza A, Desai A, Ferrero C, Germagnoli L, Mantovani A, Rescigno M, ICH COVID-19 Task-force. 2022. BNT162b2 vaccine induces antibody release in saliva: a possible role for mucosal viral protection? EMBO Mol Med 14:e15326. doi:10.15252/emmm.202115326. 35393790 PMC9081904

[36] Callow KA. 1985. Effect of specific humoral immunity and some non-specific factors on resistance of volunteers to respiratory coronavirus infection. J Hyg (Lond) 95:173–189. doi:10.1017/s0022172400062410. 2991366 PMC2129501

[37] Johansen FE, Kaetzel CS. 2011. Regulation of the polymeric immunoglobulin receptor and IgA transport: new advances in environmental factors that stimulate pIgR expression and its role in mucosal immunity. Mucosal Immunol 4:598–602. doi:10.1038/mi.2011.37. 21956244 PMC3196803

[38] Spiekermann GM, Finn PW, Ward ES, Dumont J, Dickinson BL, Blumberg RS, Lencer WI. 2002. Receptor-mediated immunoglobulin G transport across mucosal barriers in adult life: functional expression of FcRn in the mammalian lung. J Exp Med 196:303–310. doi:10.1084/jem.20020400. 12163559 PMC2193935

[39] Khoury DS, Cromer D, Reynaldi A, Schlub TE, Wheatley AK, Juno JA, Subbarao K, Kent SJ, Triccas JA, Davenport MP. 2021. Neutralizing antibody levels are highly predictive of immune protection from symptomatic SARS-CoV-2 infection. Nat Med 27:1205–1211. doi:10.1038/s41591-021-01377-8. 34002089

[40] Feng S, Phillips DJ, White T, Sayal H, Aley PK, Bibi S, Dold C, Fuskova M, Gilbert SC, Hirsch I, Humphries HE, Jepson B, Kelly EJ, Plested E, Shoemaker K, Thomas KM, Vekemans J, Villafana TL, Lambe T, Pollard AJ, Voysey M, Oxford COVID Vaccine Trial Group. 2021. Correlates of protection against symptomatic and asymptomatic SARS-CoV-2 infection. Nat Med 27:2032–2040. doi:10.1038/s41591-021-01540-1. 34588689 PMC8604724

[41] Wang Z, Lorenzi JCC, Muecksch F, Finkin S, Viant C, Gaebler C, Cipolla M, Hoffmann HH, Oliveira TY, Oren DA, Ramos V, Nogueira L, Michailidis E, Robbiani DF, Gazumyan A, Rice CM, Hatziioannou T, Bieniasz PD, Caskey M, Nussenzweig MC. 2021. Enhanced SARS-CoV-2 neutralization by dimeric IgA. Sci Transl Med 13:eabf1555. doi:10.1126/scitranslmed.abf1555. 33288661 PMC7857415

[42] Hennings V, Thörn K, Albinsson S, Lingblom C, Andersson K, Andersson C, Järbur K, Pullerits R, Idorn M, Paludan SR, Eriksson K, Wennerås C. 2022. The presence of serum anti-SARS-CoV-2 IgA appears to protect primary health care workers from COVID-19. Eur J Immunol 52:800–809. doi:10.1002/eji.202149655. 35128644 PMC9087394

[43] Sheikh-Mohamed S, Isho B, Chao GYC, Zuo M, Cohen C, Lustig Y, Nahass GR, Salomon-Shulman RE, Blacker G, Fazel-Zarandi M, Rathod B, Colwill K, Jamal A, Li Z, de Launay KQ, Takaoka A, Garnham-Takaoka J, Patel A, Fahim C, Paterson A, Li AX, Haq N, Barati S, Gilbert L, Green K, Mozafarihashjin M, Samaan P, Budylowski P, Siqueira WL, Mubareka S, Ostrowski M, Rini JM, Rojas OL, Weissman IL, Tal MC, McGeer A, Regev-Yochay G, Straus S, Gingras A-C, Gommerman JL. 2022. Systemic and mucosal IgA responses are variably induced in response to SARS-CoV-2 mRNA vaccination and are associated with protection against subsequent infection. Mucosal Immunol 15:799–808. doi:10.1038/s41385-022-00511-0. 35468942 PMC9037584

[44] Rikhtegaran Tehrani Z, Saadat S, Saleh E, Ouyang X, Constantine N, DeVico AL, Harris AD, Lewis GK, Kottilil S, Sajadi MM. 2020. Performance of nucleocapsid and spike-based SARS-CoV-2 serologic assays. PLoS One 15:e0237828. doi:10.1371/journal.pone.0237828. 33137138 PMC7605638

[45] Keech C, Albert G, Cho I, Robertson A, Reed P, Neal S, Plested JS, Zhu M, Cloney-Clark S, Zhou H, Smith G, Patel N, Frieman MB, Haupt RE, Logue J, McGrath M, Weston S, Piedra PA, Desai C, Callahan K, Lewis M, Price-Abbott P, Formica N, Shinde V, Fries L, Lickliter JD, Griffin P, Wilkinson B, Glenn GM. 2020. Phase 1–2 trial of a SARS-CoV-2 recombinant spike protein nanoparticle vaccine. N Engl J Med 383:2320–2332. doi:10.1056/NEJMoa2026920. 32877576 PMC7494251

[46] Mateus J, Dan JM, Zhang Z, Rydyznski Moderbacher C, Lammers M, Goodwin B, Sette A, Crotty S, Weiskopf D. 2021. Low-dose mRNA-1273 COVID-19 vaccine generates durable memory enhanced by cross-reactive T cells. Science 374:eabj9853. doi:10.1126/science.abj9853. 34519540 PMC8542617

