# Supplementary figures and images for: Mucosal and Systemic Responses to Severe Acute Respiratory Syndrome Coronavirus 2 Vaccination Determined by Severity of Primary Infection
Source: mSphere. 2022 Nov 2;7(6):e00279-22. doi: 10.1128/msphere.00279-22 (PMC9769618; doi:10.1128/msphere.00279-22)

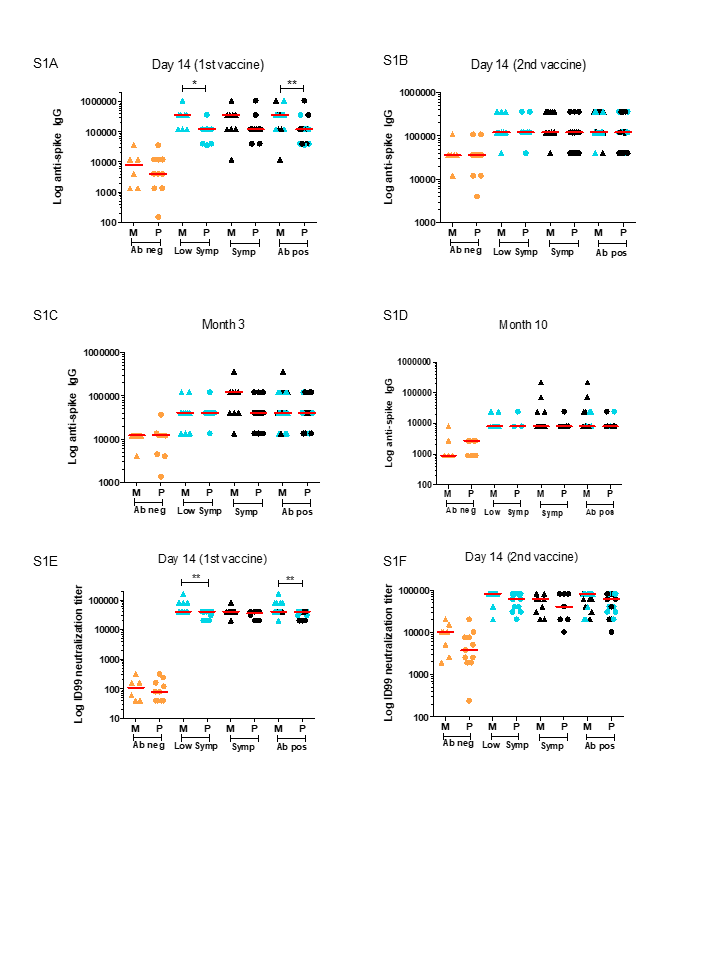

Supplement: FIG S1 [file msphere.00279-22-s0001.tif]

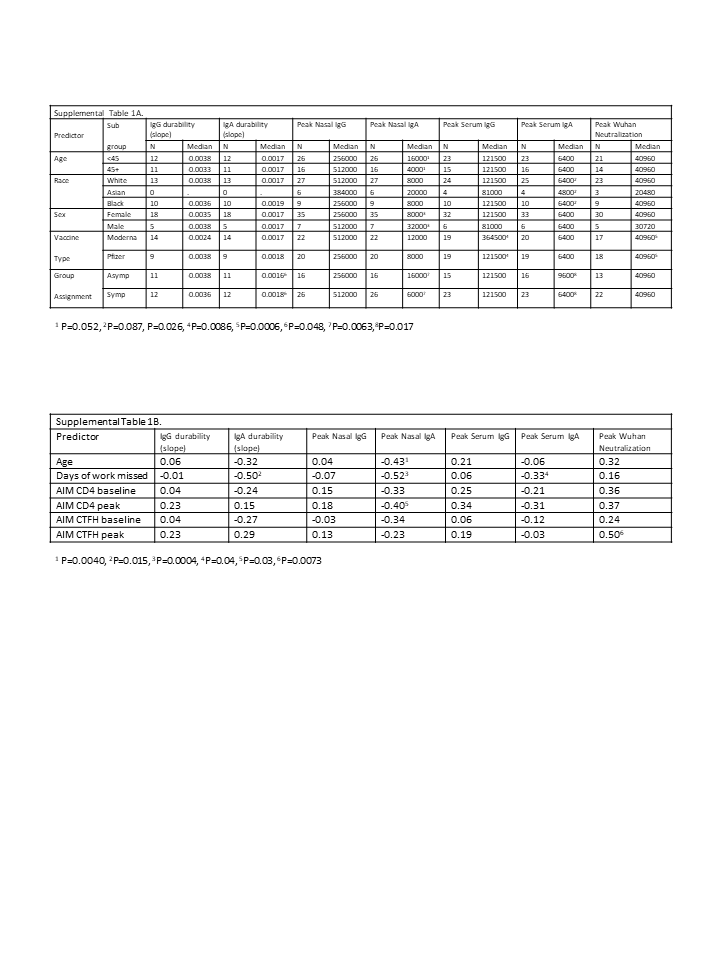

Supplement: TABLE S1 [file msphere.00279-22-s0010.tif]

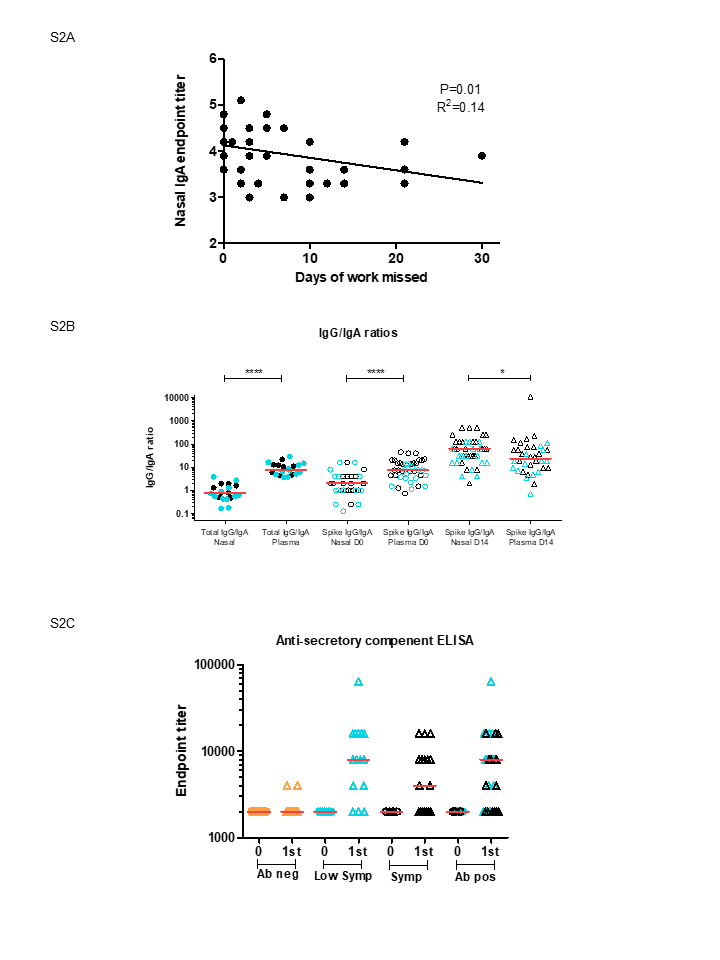

Supplement: FIG S2 [file msphere.00279-22-s0002.tif]

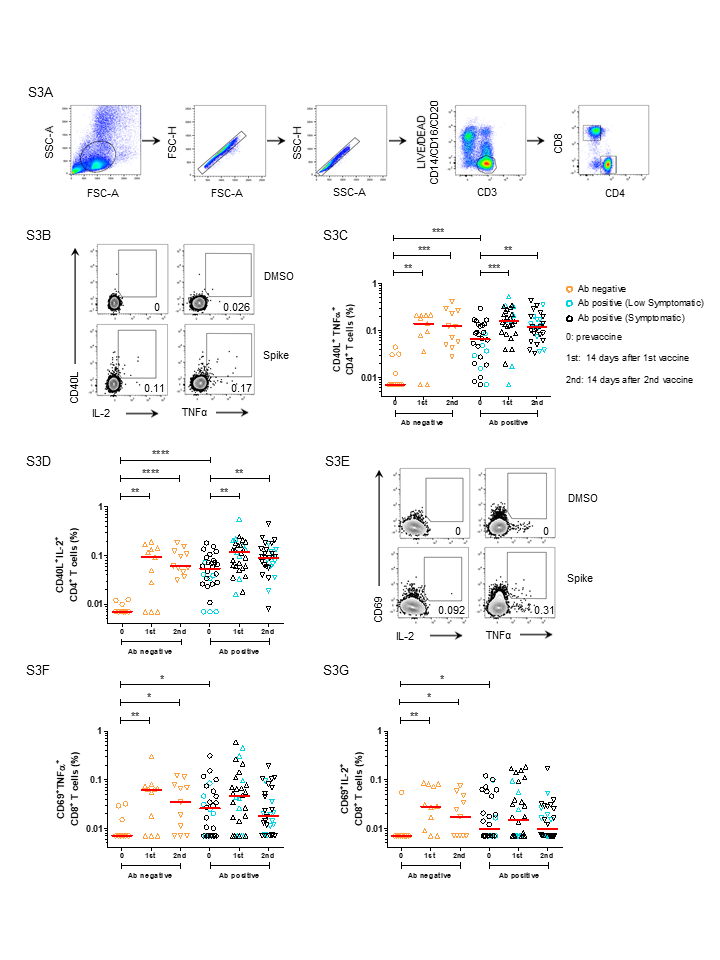

Supplement: FIG S3 [file msphere.00279-22-s0003.tif]

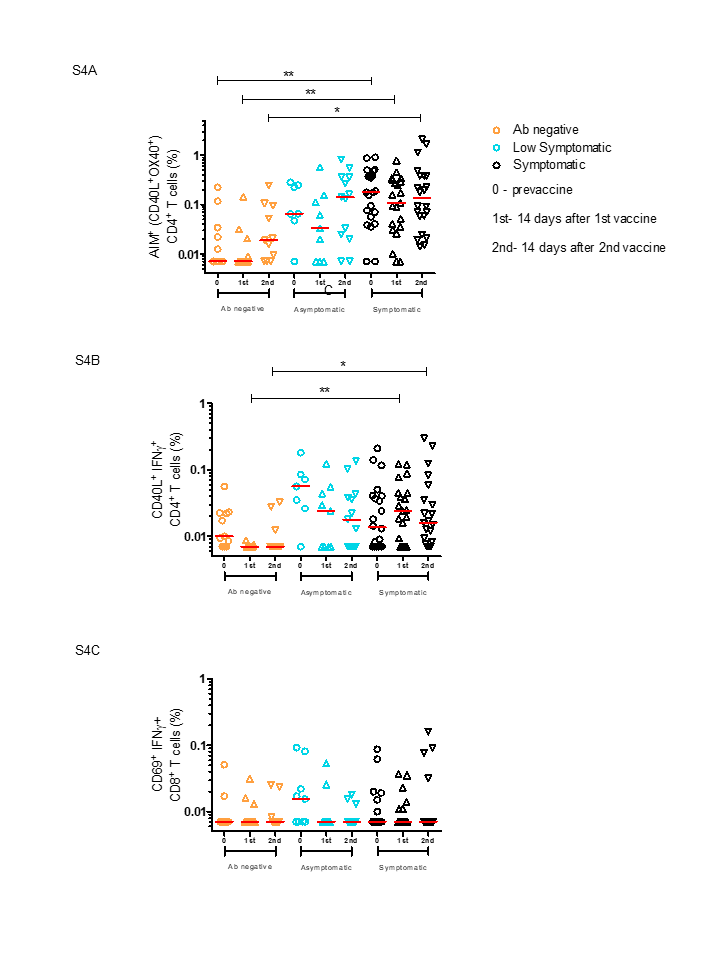

Supplement: FIG S4 [file msphere.00279-22-s0004.tif]

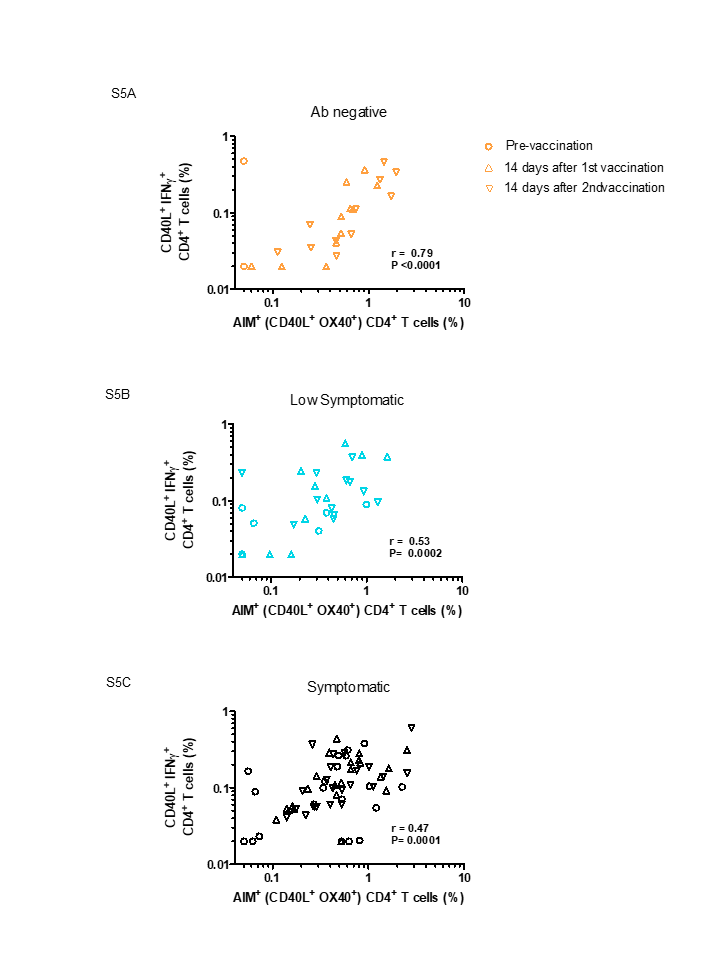

Supplement: FIG S5 [file msphere.00279-22-s0005.tif]

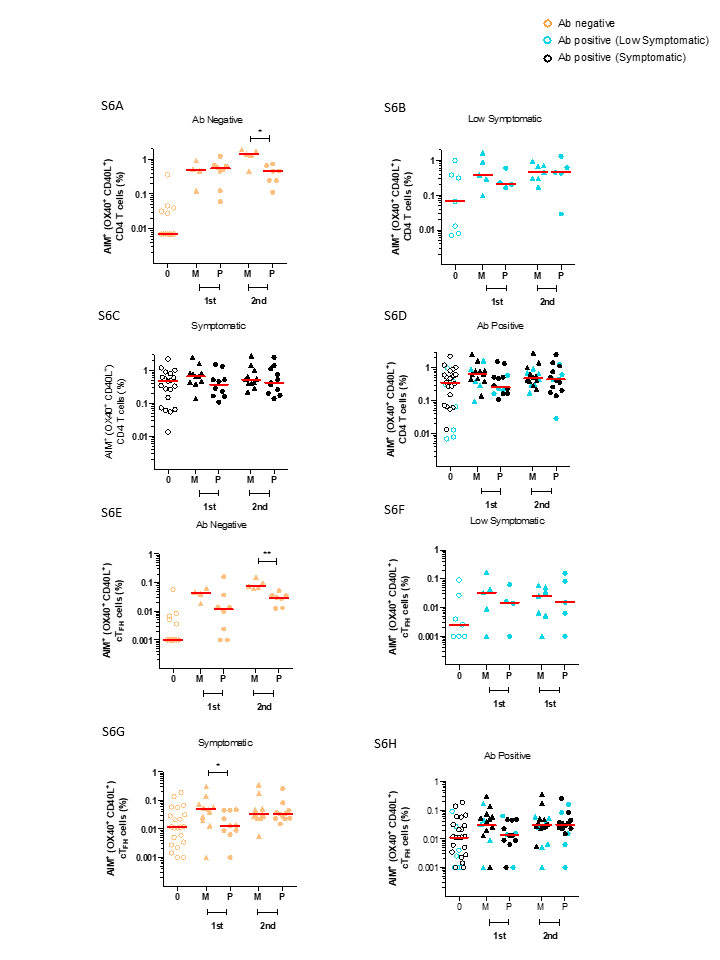

Supplement: FIG S6 [file msphere.00279-22-s0006.tif]

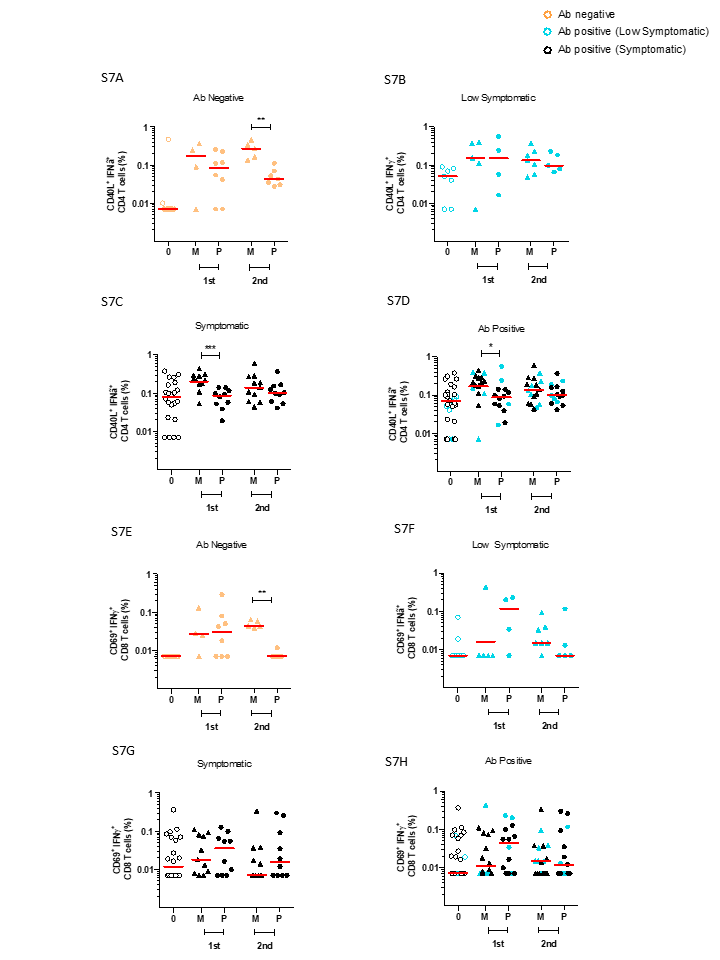

Supplement: FIG S7 [file msphere.00279-22-s0007.tif]

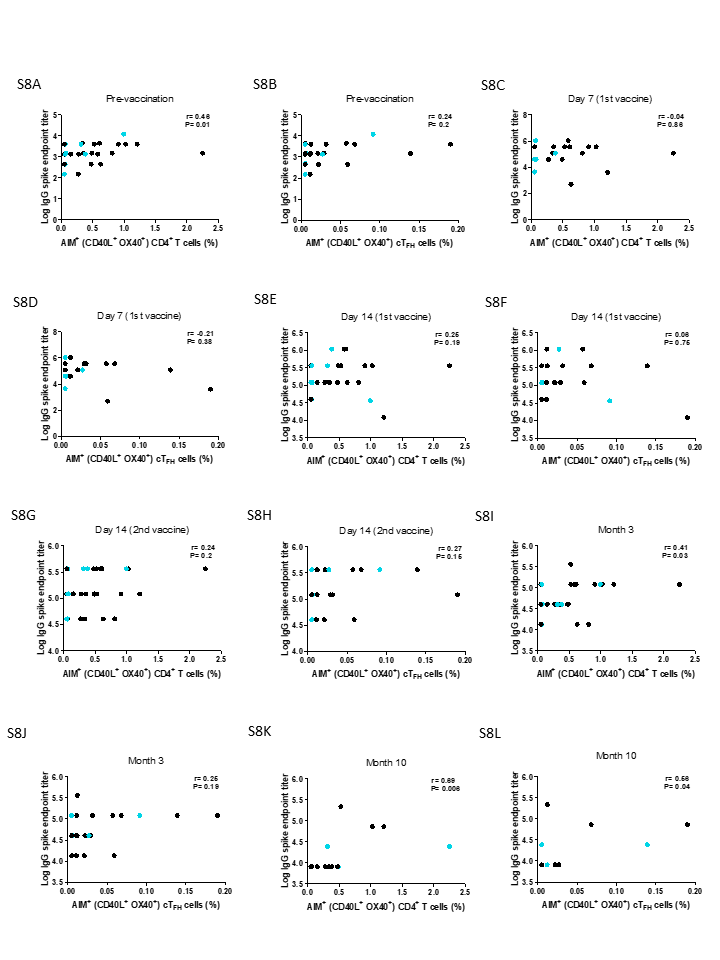

Supplement: FIG S8 [file msphere.00279-22-s0008.tif]

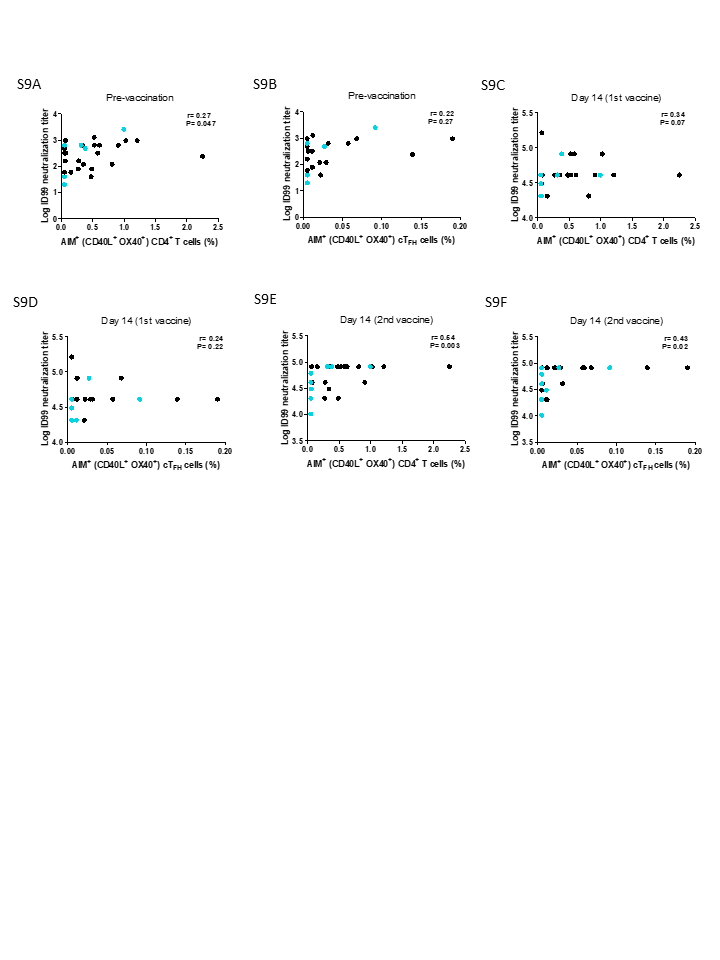

Supplement: FIG S9 [file msphere.00279-22-s0009.tif]
